# Follow-up after major traumatic injury: a survey of services in Australian and New Zealand public hospitals

**DOI:** 10.1186/s12913-024-11105-w

**Published:** 2024-05-15

**Authors:** Elizabeth Wake, Jamie Ranse, Don Campbell, Belinda Gabbe, Andrea P. Marshall

**Affiliations:** 1 Trauma Service, Gold Coast University Hospital, Gold Coast, QLD Australia; 2 School of Medicine and Dentistry, Griffith University, Gold Coast, Australia; 3 School of Nursing and Midwifery, Griffith University, Gold Coast, Australia; 4 Menzies Health Institute Queensland, Griffith University, Gold Coast Campus, Gold Coast, QLD Australia; 5 School of Public Health and Preventative Medicine, Monash University, Melbourne, VIC Australia; 6 Midwifery Education and Research Unit, Gold Coast University Hospital, Nursing, QLD Australia

**Keywords:** Major trauma, Content validity, Donabedian, Follow-Up, Post discharge, Trauma clinic, Trauma centers, Traumatic Injury, Survey

## Abstract

**Background:**

Increased survival from traumatic injury has led to a higher demand for follow-up care when patients are discharged from hospital. It is currently unclear how follow-up care following major trauma is provided to patients, and how, when, and to whom follow-up services are delivered. The aim of this study was to describe the current follow-up care provided to patients and their families who have experienced major traumatic injury in Australia and New Zealand (ANZ).

**Methods:**

Informed by Donabedian’s ‘Evaluating the Quality of Medical Care’ model and the Institute of Medicine’s Six Domains of Healthcare Quality, a cross-sectional online survey was developed in conjunction with trauma experts. Their responses informed the final survey which was distributed to key personnel in 71 hospitals in Australia and New Zealand that (i) delivered trauma care to patients, (ii) provided data to the Australasian Trauma Registry, or (iii) were a Trauma Centre.

**Results:**

Data were received from 38/71 (53.5%) hospitals. Most were Level 1 trauma centres (*n* = 23, 60.5%); 76% (*n* = 16) follow-up services were permanently funded. Follow-up services were led by a range of health professionals with over 60% (*n* = 19) identifying as trauma specialists. Patient inclusion criteria varied; only one service allowed self-referral (3.3%). Follow-up was within two weeks of acute care discharge in 53% (*n* = 16) of services. Care activities focused on physical health; psychosocial assessments were the least common. Most services provided care for adults and paediatric trauma (60.5%, *n* = 23); no service incorporated follow-up for family members. Evaluation of follow-up care was largely as part of a health service initiative; only three sites stated evaluation was specific to trauma follow-up.

**Conclusion:**

Follow-up care is provided by trauma specialists and predominantly focuses on the physical health of the patients affected by major traumatic injury. Variations exist in terms of patient selection, reason for follow-up and care activities delivered with gaps in the provision of psychosocial and family health services identified. Currently, evaluation of trauma follow-up care is limited, indicating a need for further development to ensure that the care delivered is safe, effective and beneficial to patients, families and healthcare organisations.

**Supplementary Information:**

The online version contains supplementary material available at 10.1186/s12913-024-11105-w.

## Background

Improved survival following traumatic injury has led to increased demand for trauma follow-up care when patients are discharged from hospital. It is estimated that approximately 50% of all patients who experience physical trauma will require some form of follow-up care after hospital discharge [1]. Almost a quarter of patients continue to have significant health issues after experiencing traumatic injury [2]. Injured patients have greater use of health services after discharge from hospital when compared to the general population and this resource use can remain elevated for several years after injury [3] resulting in increased healthcare expenditure [4]. 

In high income countries, the development and introduction of trauma systems, which are integrated, and systematic structures designed to facilitate and coordinate a system response to provide optimal care to injured patients [5], has led to a decrease in mortality and disability of individuals affected by traumatic injuries [6, 7]. In Australia, trauma systems are organised into a hub and spoke model; level 1 trauma centres are the ‘hub’ and contain the full spectrum of resources and specialties to care for the most critically injured patients, whilst levels 2 to 4 (spokes), have a graduated decrease in their trauma care capabilities resulting in level 4 trauma centers that can provide early resuscitation, stabilisation and transfer of patients to higher levels of trauma care [8]. (Additional File 1) This centralization of patient care facilitates the most injured patients to the most appropriate level of care [9]. 

The classification of trauma centre level is underpinned by evidence-based consensus standards published by professional organisations [8, 10]. These standards provide guidance on the components necessary to achieve optimal trauma care from the pre-hospital environment to rehabilitation and follow-up care. Whilst the Australian standards recommend the inclusion of follow-up care as part of comprehensive trauma care, the trauma verification standards are silent on how follow-up clinics should be implemented or how evaluation of the clinic should be undertaken [8]. 

In a recent scoping review [11] wide variations in how follow-up services for patients with major trauma are provided were identified and ranged from delivering ‘routine’ follow-up care, such as maintaining contact and/or re-examination of patients following hospital treatment [12], to purpose designed recovery programs, such as the Trauma Survivors Network (TSN) [13]. Gaps in relation to how and why follow-up services are established were identified [11]. Of the studies included, most were from outside of Australia and New Zealand and given differences in how health services are provided, the findings of these studies may have limited application in other healthcare contexts.

The diverse approaches to follow-up care following traumatic injury raise questions about service provision, the quality of care provided and how the outcomes are achieved for patients and their families, and for health care organisations. An evaluation of trauma follow-up care, using quality frameworks such as Donabedian ‘Evaluating the Quality of Medical Care’ model or the Institute of Medicine’s’ Six Domains of Healthcare Quality’ (IOM), is needed [14]. However, before an evaluation can occur, an understanding of the types of trauma follow-up services that are available is first required.

## Methods

### Aim

The aim of this study is to report on existing trauma follow-up service provision in Australia and New Zealand. This was achieved by:

I. Developing, with trauma experts, a valid survey to describe trauma follow-up services.
II. Describing the current follow-up care provided to patients and their families who have experienced major traumatic injury in Australia and New Zealand.

### Research question

What follow-up services for patients who have experienced major trauma are currently provided in Australia and New Zealand?

### Design

This study was a prospective quantitative web-based survey. The Checklist for Reporting Results of Internet E-Surveys (CHERRIES) was followed when reporting this study [15]. (Additional File 2).

### Content validity instrument development

The initial survey was informed using the findings from an earlier scoping review [11] and the conceptual framework which was informed by Donabedian [16] and the Institute of Medicine (IOM) [17] quality frameworks (Additional File 3). The Donabedian model assumes that healthcare quality is based on three domains: structure – the context where care is delivered; process – the combination of actions that make up service delivery and outcomes – the effects of the healthcare. The IOM model includes six aims for healthcare systems which include safety, effectiveness, timely, efficient, equitable and patient centered. The combining of these two frameworks has previously been used within trauma literature to identify and create quality indicators in trauma care [18]. 

The initial survey consisted of 70 survey items with questions specific to the domains of both quality frameworks (Additional File 4).

#### Sample – trauma experts

A purposive sampling technique [19] was used to identify medical, nursing, and allied health clinicians who were considered experts in providing care to patients with major trauma. Trauma experts were identified through established trauma networks such as the Australian and New Zealand Trauma Society and Australian New Zealand Trauma Registry. The identified sample included representatives from each state and territory within Australia, and New Zealand.

#### Content validity testing

Content validity is defined as the degree to which an instrument (or assessment) has an appropriate sample of items for the construct (or topic) being measured (assessed) [20]. While the survey was not designed to ‘measure’ a particular construct, the general concept of content validity was important to the development of survey items to ensure that data collected were comprehensive and appropriate to the topic. Trauma experts were asked to rate each survey item for relevance, using a scale from 1 (not relevant) to 4 (very relevant). Free text comment boxes were provided where participants could elaborate on survey item(s) and comment on response options.

#### Data collection

The content validity assessment was conducted between January 2023 and February 2023. Study data were collected and managed using REDCap (Research Electronic Data Capture) electronic data capture tool [21, 22]. REDCap is a secure, web-based software platform designed to support data capture for research studies, providing (1) an intuitive interface for validated data capture; (2) audit trails for tracking data manipulation and export procedures; (3) automated export procedures for seamless data downloads to common statistical packages; and (4) procedures for data integration and interoperability with external sources.

Potential participants, identified through professional networks by members of the research team, were sent a survey invitation via email by the study principal investigator, which contained details about the study and completion instructions and the electronic link that could be accessed through any device. A four-week period was allowed for completion, with weekly reminder emails.

#### Data analysis

A Content Validity Index (CVI) was calculated to measure the preliminary survey and was obtained by calculating the number of responses rated “3” or “4” and dividing this number by the total number of participants. The accepted minimum mean I-CVI of 0.78 was used in this study, irrespective of the number of participants and in accordance with I-CVI principles [23]. Items that scored an I-CVI of less than 0.78 and/or received written feedback were discussed by the research team for relevance and a decision made about their inclusion in the final survey. The results from the content validity assessment can be found in Additional File 5.

### Final instrument

#### Setting and sample

This cross-sectional online survey was undertaken with trauma healthcare clinicians who work at public hospitals throughout Australia and New Zealand that deliver care to patients affected by major traumatic injuries. Hospitals needed to meet one of the following criteria to be eligible to participate: [1] an accredited trauma centre (currently or previously) according to the Royal Australasian College of Surgeons (RACS); [2, 8] a regional trauma centre; [3] a trauma service, or [4] submit data to the Australian New Zealand Trauma Registry (ATR) [5]. 

#### Survey instrument

The final version of the survey contained 50 survey items divided into four sections: Hospital Demographics, and the Donabedian [16] and the Institute of Medicine (IOM) [17] quality frameworks domains (Additional File 6).

#### Data collection

The final survey was conducted between April and May 2023. Potential participants at each eligible institution, identified through professional networks by members of the research team, were sent a survey invitation via email which contained a description of the study and a link to access and complete the on-line survey. A total of 71 hospitals were included. Participants were encouraged to recommend an alternative point of contact or key person if they were unable to complete the survey on behalf of their hospital. A reminder was sent weekly for 4 weeks after which the survey was closed.

#### Data analysis

On completion of the allocated survey time, the survey responses were reviewed for completeness. The data was then extracted from REDCap and any identifiable variables (Hospital name, State, Country) were re-coded and allocated a unique study number. Missing data were treated using a case analysis (listwise deletion) approach, where cases are removed from the sample if they had missing data on any of the variables in the analysis to be conducted [24]. The data were then imported into STATA 15 (College Station, TX, USA) in preparation for analysis.

Descriptive analysis using frequencies and percentages was used to describe the response rates, survey completion rates, and hospital demographics for all survey responses. Descriptive statistics such as means with standard deviations, medians with IQR, and frequencies with percentages were used to summarise survey results.

To detect associations between categorical variables (countries, trauma centres and follow-up service delivery components) a Chi-Square test was used. To detect associations between follow-up funding status and country, and care activities and health discipline a logistic regression model was used. Results are presented as odds ratios (OR) with 95% confidence intervals (CI). P-values of < 0.05 was considered statistically significant.

For survey responses with ‘free text’ responses, manifest deductive content analysis was performed.

#### Ethics

The study was approved by the Human Research Ethics Committee at Griffith University (GU 2022/839) and by Gold Coast Hospital and Health Services (HREC/2023/QGC/94,879) under the National Mutual Acceptance scheme. An implied consent process was requested and approved by both ethics’ committees.

## Results

Participants from thirty-eight hospitals responded to the survey (54%), with 30 completing the online survey and eight providing information via email; of the ones that provided information by email 75% (*n* = 6) identified as a regional hospital.

### Structure: the context in which care is delivered

#### Physical facility, equipment, and resources

Most respondents were from tertiary hospitals (*n* = 25, 66%), with 61% (*n* = 23) identifying as level 1 trauma centres (Table 1); 40% (*n* = 12) of these level 1 trauma centers were currently verified at the time of the survey in accordance with the Royal College of Surgeons (ANZ) verification criteria [18]. In both Australia and New Zealand, trauma follow-up services had access to equipment such as electronic medical records (*n* = 25, 83%) and radiology and pathology reports (*n* = 29, 97%); less than half (*n* = 13, 43%) could access electronic medical records outside their health service and were predominantly located in Australia (*n* = 10, 77%). Over three-quarters of follow-up services at Level 1 trauma centres had permanent funding (*n* = 16, 76%); 40% (*n* = 12) stated that they did not have secure funding or were unsure of the funding status. Permanency of follow-up service funding was significantly associated with the type of hospital, with tertiary hospitals associated with a sevenfold increase in the odds of having permanent funding (OR 7.08, CI 1.07–46.7, *p* 0.042). Trauma follow-up services in New Zealand were associated with increased odds of permanent funding however this was not statistically significant (OR 1.2, CI 0.18–7.92, *p* 0.850). The majority of follow-up services had a dedicated follow-up clinic space (*n* = 18, 60%); 87% (*n* = 26) indicated telehealth was also available.

**Table 1:** Physical characteristics by country

	All *N* = 38	Australia *N* = 30	New Zealand *N* = 8
Patient Population			
- Adult - Paediatrics - Both	9 (24%) 6 (16%) 23 (61%)	8 (27%) 5 (17%) 17 (57%)	1 (12.5%) 1 (12.5%) 6 (75%)
Type of Hospital			
- Tertiary - Regional - Rural	25 (66%) 13 (34%) 0	19 (63%) 11 (37%) 0	6 (75%) 2 (25%) 0
Trauma Centre Level*			
- 1 - 2 - 3 - 4	23 (61%) 1 (2.5%) 13 (34%) 1 (2.5%)	17 (57%) 1 (3%) 11 (37%) 1 (3%)	6 (75%) 0 2 (25%) 0
Survey Data Only	*N* = 30	*N* = 24	*N* = 6
Permanently Funded:			
- Yes - No - Unsure	18 (60%) 11 (37%) 1 (3%)	14 (58%) 9 (38%) 1 (4%)	4 (67%) 2 (33%) 0
Location:			
- Same hospital - Different location - Telehealth only	26 (87%) 2 (6.5%) 2 (6.5%)	22 (92%) 1 (4%) 1 (4%)	4 (67%) 1 (16.5%) 1 (16.5%)
Dedicated Clinic Space			
- Yes - No - Not answered	18 (60%) 7 (23%) 5 (17%)	16 (53%) 5 (17%) 9 (30%)	2 (33.3%) 2 (33.3%) 2 (33.3%)

* Royal Australasian College of Surgeons Trauma Verification Level

#### Staff

**Fig. 1:**
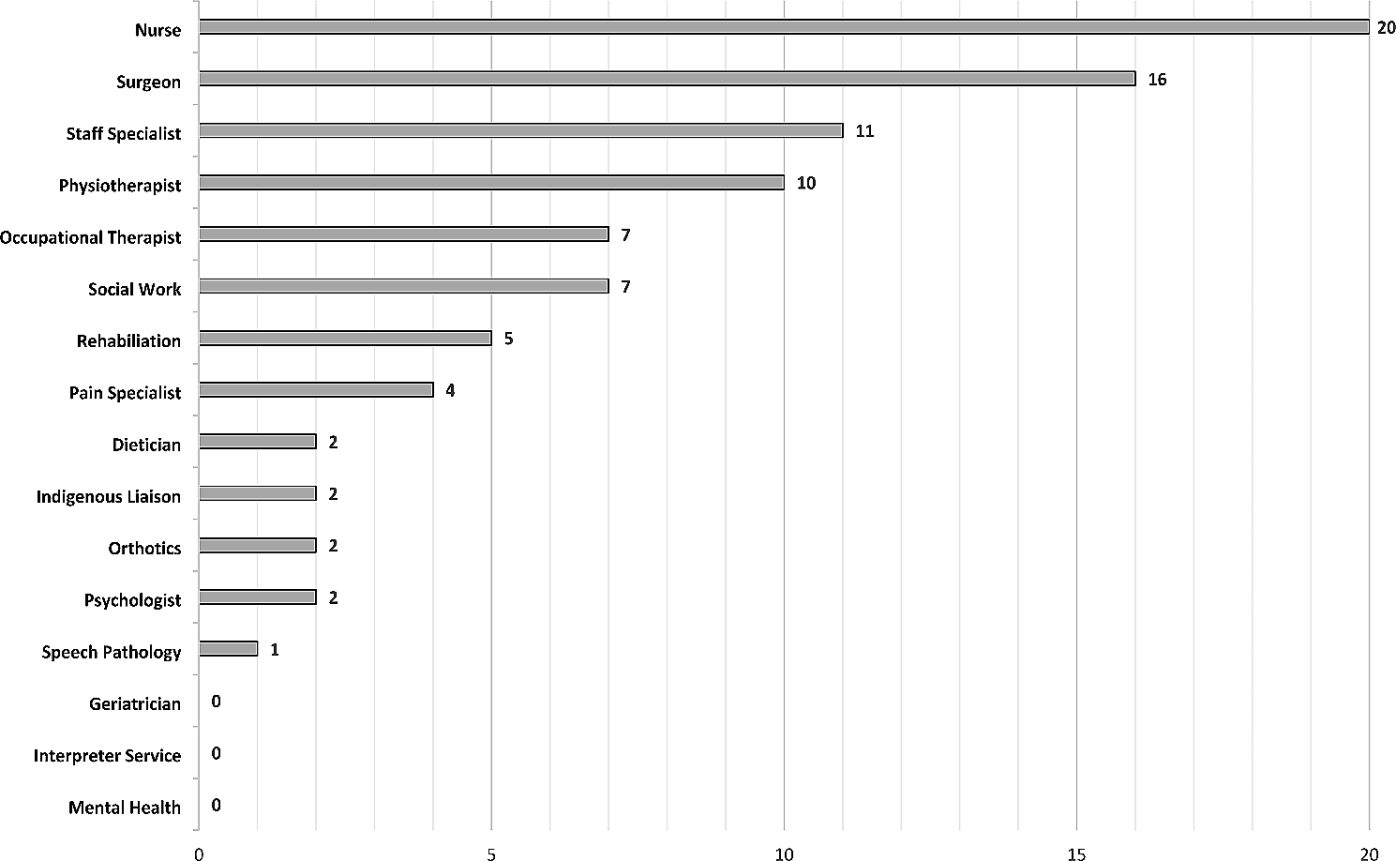
Health professional disciplines that work in trauma follow up services through Australia and New Zealand

Follow-up services were led by all health professionals of the multi-disciplinary team with over 60% (*n* = 19) identifying as trauma specialists. Follow-up services in Level 1 trauma centres had a wider range of health disciplines who regularly delivered care, including rehabilitation services (*n* = 5, 16.7%), social workers (*n* = 7, 23.3%) and pain management (*n* = 4, 13.3%) (Fig. 1). No follow-up service had staff who specialised in geriatrics, mental health or interpreter services.

### Process: how care is delivered

#### Who, why, activities, frequency and timing

Inclusion criteria through which trauma follow-up services utilised to identify patients were varied and ranged from patients with major traumatic injuries only (*n* = 4, 13%) to patients on specified injury pathways (*n* = 10, 33%); 30% (*n* = 9) of follow-up services had ‘all patients’ criteria (Table 2). Over half of the follow-up services (n = 16, 53%) were provided to patients within 2 weeks following hospital discharge and included both ‘routine’ and specific ongoing care (n = 20, 67%); 37% (n = 11) of follow-up services offered multiple appointments if required.

**Table 2:** Who, why, when, by health discipline

	All *N* = 30	Nurse Led *N* = 9	Doctor Led *N* = 12	MDT† Led *N* = 9
Who (n, %) Inclusion Criteria*:				
- All trauma patients - Major trauma only - Specific Inc/Exclusion - Injury pathway - GP referral - Health service referral - Self-referral - Family referral - Dependent on proximity	9 (30%) 4 (13%) 5 (17%) 10 (33%) 3 (10%) 15 (50%) 1 (3%) 0 2 (7%)	2 (22%) 3 (33%) 2 (22%) 3 (33%) 1 (11%) 3 (33%) 0 0 0	3 (25%) 0 1 (8%) 2 (17%) 1 (8%) 7 (58%) 0 0 1 (8%)	4 (44%) 1 (11%) 2 (22%) 5 (56%) 1 (11%) 5 (56%) 1 (11%) 0 1 (11%)
Why (n, %) Rationale*				
- Routine - Specific ongoing care - Emotional/Psychological - Protocolised care	20 (67%) 20 (67%) 9 (30%) 5 (17%)	5 (56%) 7 (78%) 4 (44%) 2 (22%)	8 (67%) 9 (75%) 9 (75%) 3 (25%)	7 (78%) 4 (44%) 2 (22%) 0
When (n, %) Frequency				
- Daily - Everyday - 3–4 times per week - 1–2 times per week - Weekly - Fortnightly	5 (17%) 0 1 (3%) 3 (10%) 8 (27%) 4 (13%)	1 (11%) 0 0 1 (11%) 4 (44%) 1 (11%)	2 (17%) 0 0 2 (17%) 1 (8%) 3 (25%)	2 (22%) 0 1 (11%) 3 (33%) 0 0
Timepoints (n, %)				
- Within 2 weeks of dc - Within 3–4 weeks of dc - Within 5–6 weeks of dc - Within 7–8 weeks of dc - > 8 weeks	16 (53%) 5 (17%) 1 (3%) 0 0	4 (44%) 3 (33%) 0 0 0	6 (50%) 1 (8%) 1 (8%) 0 0	6 (67%) 1 (11%) 0 0 0
Discharge criteria (n, %)				
- Yes - No - Unsure	4 (13%) 19 (63%) 7 (23%)	2 (22%) 7 (78%) 0	0 6 (50%) 6 (50%)	2 (22%) 6 (67%) 1 (11%)

* Multiple responses permitted; † Multi-Disciplinary Team

**Fig. 2:**
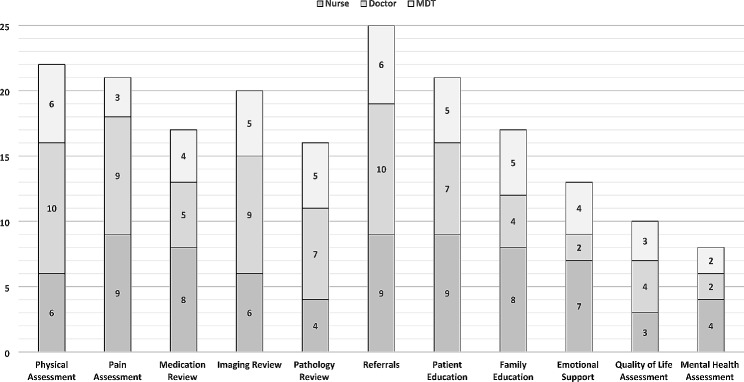
Activities performed at trauma follow up services by health discipline MDT ? Multi Disciplinary Team

Care activities undertaken at the follow-up services focused largely on the physical health of patients (Fig. 2). Psychosocial activities, such as mental health and quality of life assessments and the provision of emotional support to patients and families were reported but were the least common activities performed. In follow-up services that were predominantly medically led, there were significantly decreased odds of the provision of emotional support (OR 0.89, p 0.01, CI 0.006–0.508) than services led by other disciplines.

The types of psychosocial assessments performed included screening for Post-Traumatic Stress Disorder (PTSD) (*n* = 5, 16.7%), anxiety (*n* = 7, 23.3%) and depression (*n* = 7, 23.3%). Only eight follow-up services (23.5%) provided a psychosocial assessment; no paediatric only follow-up services, undertook anxiety and/or depression assessments with either the patients or the family.

Two paediatric follow-up clinics indicated that services were provided for family members of patients; only one follow-up clinic included access to a social worker. Despite most hospitals (60.5%, *n* = 23) providing care for both adults and paediatric trauma patients, none of these hospitals (*n* = 23) indicated that they provided services for family members.

### Outcomes: results of healthcare delivery

Evaluation of follow-up services was largely in the form of patient and family satisfaction surveys. Administration of the surveys were either part of a health service initiative (patient *n* = 3, 20%; family *n* = 5, 38%) or follow-up service specific (patient *n* = 3, 20%; family *n* = 1, 8%). Survey responses were low with only 50% (*n* = 15) of sites responding yes to patient satisfaction surveys and 43% (*n* = 13) to family satisfaction surveys. Follow-up service delivery outcomes were measured by only one site in the form of key performance indicators which included attendance rates and the percentage of patients that complete follow-up compared to percentage of patients lost to follow-up.

## Discussion

In this bi-national survey of follow-up services provided to patients following major traumatic injury, we identified wide variations in how health care is delivered. Whilst follow-up services provided by trauma specialists were reported to be widely available, there appears to be important gaps in the availability and accessibility of psychological support, family centered healthcare services and longer-term trauma follow-up care; health service evaluation, to determine the quality and safety of the care delivered, is lacking in the majority of follow-up services.

### Psychological support

Access to specialist psychological care was included by only one trauma follow-up service, who included a permanent psychologist as part of the regular clinical team to deliver follow-up care. Traumatic injury can significantly impact emotional well-being, with nearly 50% of trauma survivors experiencing depression, anxiety, or acute stress disorder [25, 26]. These psychological effects can negatively impact physical health outcomes [27–32] and an individual’s perception of recovery [33, 34]. Understanding and identifying psychological factors can help identify targeted interventions to improve patient health outcomes [35]. Within the literature, the need for awareness and attention to be paid to the emotional health of patients affected by traumatic injury is extensive; however, only a quarter of follow-up services reported incorporating a formal assessment of psychological health. Whilst this could relate to the lack of psychological specialists within the multi-disciplinary follow-up team, psychological care can also be accessed through primary care providers under government led initiatives such as the ‘Better Access Initiative’ [36] and the “Very Low Cost” access scheme [37]. However, the accessibility and availability of psychological care providers can be dependent upon the country and the geographical location that the patient resides in [38], in addition to the socio-economic status of the patient as, although the cost of accessing the psychological care services can be subsidized under government initiatives, it is not always at no cost to the patient. In their evaluation of a trauma recovery program, the Trauma Collaborative Care, Wegener et al [39] identified barriers cited by clinicians in providing psychological care which included lack of time, resources, skills and clinician confidence. Whilst training clinicians in the use of screening tools to identify patients with potential emotional problems can help with some of these issues, this does not mitigate the need for the ongoing resources required to deliver this component of care to support patients who have been identified [40]. 

### Family and social support

The role of social workers within the area of trauma care has been found to be highly valued by both patients, family members and clinicians; [41] yet the inclusion of a social worker in trauma follow-up care delivery was limited. Social support plays an essential and important role in the delivery of emotional, informational and functional resources [42], such as financial and legal guidance following traumatic injury for both patients and family members. Importantly, there are a reduction in psychological symptoms of post-traumatic stress disorder PTSD, depression and anxiety when patients and families perceive they have access to social support, and this is strongly correlated with physical and mental health outcomes at 6- and 12-months post injury [42]. 

A patients’ social support network will frequently be relied upon to provide ongoing care and recovery to the trauma patient, often in the role of an informal caregivers. The informal care giver role can have consequences with caregivers experiencing increased psychological distress and decreased resilience [43]. This is especially relevant within the paediatric trauma population where the caregivers, usually the parents, often experience increased feelings of guilt and responsibility [42, 44, 45]. Whilst the majority of respondents to the survey provided trauma care to both the adult and paediatric populations, no follow-up service identified that caregivers could access support through the follow-up service.

### Trauma recovery programs

A solution to address the barriers of delivering psychological care post traumatic injury whilst encompassing the advantages of social support, is the implementation of trauma recovery programs, such as the Trauma Survivor Network (TSN) [46]. These programs, delivered by the multidisciplinary team from a trauma centre, empower patients to take charge of their recovery by increasing resilience and self-efficacy and addressing mental health and social needs by using a patient and family centered care approach [47]. Benefits include their longitudinal structure which aligns with the recovery journey following major trauma; reduction in the incidence of depression and PTSD rates; [48] increased patient and family satisfaction with care; [49] and improved adherence to post-operative care plans and out-patient appointments as well a decrease emergency department presentation. Level 1 trauma centres within the USA are now required to implement a recovery program as a standard of care [10]. However, the positive benefits of recovery programs should be interpreted with caution as they are not consistently predictable [50]. Additionally, recovery programs outside of the America’s are rare; the benefits have yet to be validated in other healthcare settings where the structure and delivery of both in-hospital and primary care are different, making extrapolation and generalisability of the findings challenging.

### Future of trauma follow-up care

Despite the identified gaps in the current health service delivery of trauma follow up care within Australia and New Zealand, the fact that follow-up care exists, is accessible for patients and is provided by trauma specialists is encouraging. Trauma recovery programs can help to bridge the gaps between physical and psychological health needs, and although the evidence base is limited, the principles of recovery programs in terms of a holistic, longitudinal multi-disciplinary approach should be considered when planning any future trauma follow-up care program. Additionally, the question of whether a recovery program should be incorporated into existing follow-up services delivered by trauma centres, be a separate entity to the current ‘clinical’ follow-up or be delivered by primary care remains unanswered. An integrated approach which encompasses primary care and trauma specialists could provide an optimal model that supports the ongoing complex and longitudinal needs of both patients, families, health care organisations and primary care networks who deal with major traumatic injuries. This could be embedded within individual state’s, territory’s or a country’s pre-existing government insurance schemes such as the ‘Transport Accident Commission’ in Victoria, Australia [51] and the ‘Accident Compensation Corporation’ in New Zealand [52]. This integration may also minimise the reports from patients, families and primary care who currently feel overwhelmed and unsupported in the current system [7, 53]. Importantly, embedding these principles within trauma verification standards provides the ability to benchmark and evaluate follow-up care more effectively. Currently, evaluating follow-up care in ANZ is limited and often linked to generic health service evaluation initiatives, making targeted results challenging. As a result, it currently remains unclear how effective current trauma follow-up care is at providing high quality outcomes for both patients, families, and healthcare organisations.

### Limitations

There were some limitations to this study. Only half of the participants invited to participate responded. Additionally, although every effort was made to obtain a survey response from each state and territory within Australia and New Zealand not all responded. As this is an Australian and New Zealand study, care should be taken when using the results for a wider comparison as healthcare contexts will differ. This survey focused only on the delivery of trauma follow-up care provided by the hospital and therefore, access to psychological and family support services may be available in the primary care networks and could account for the gaps identified. Lastly whilst the use of formal assessment tools to assess emotional issues or quality of life was found to be low, there is the possibility that informal assessment occurred; this was not covered by the survey questions.

## Conclusion

This study surveyed the current provision of trauma follow-up care in Australia and New Zealand. The results indicate that follow-up care is provided by trauma specialists and predominantly focuses on the physical health of the patients affected by major traumatic injury. Variations exist in terms of patient selection, reason for follow-up and care activities delivered with gaps in the provision of psychosocial and family health services identified. Currently, evaluation of trauma follow-up care is absent, indicating a need for further development within this area to ensure the care delivered is safe, effective and beneficial to patients, families and healthcare organisations. An integrated longitudinal approach to trauma follow-up care, which enables benchmarking of patient outcomes and includes primary care networks in trauma systems, should be considered.

### Electronic supplementary material

Below is the link to the electronic supplementary material.

Supplementary Material 1

Supplementary Material 2

Supplementary Material 3

Supplementary Material 4

Supplementary Material 5

Supplementary Material 6

## Data Availability

All data generated or analysed during this study are included in this published article [and its supplementary information files].

## Abbreviations

RACS: Royal Australasian College of Surgeons
ATR: Australia New Zealand Trauma Registry
REDCap: Research Electronic Data Capture
CVI: Content Validity Index
IOM: Institute of Medicine
TSN: Trauma Survivors Network
CHERRIES: Checklist for Reporting results of Internet E–Surveys
HREC: Human Research Ethics Committee
