# Supplementary material for: Follow-up after major traumatic injury: a survey of services in Australian and New Zealand public hospitals
Source: BMC Health Serv Res. 2024 May 15;24:630. doi: 10.1186/s12913-024-11105-w (PMC11097478; doi:10.1186/s12913-024-11105-w)
Supplement: Supplementary file 1 — Supplementary Material 1 [file 12913_2024_11105_MOESM1_ESM.docx]

**Additional File 1: Trauma Centre Levels**

|  | Description  (Australia & NZ) ^(13)^ |
| --- | --- |
| Accreditation Level | **I** |
|  | - Capable of providing the full spectrum of care for the most critically injured patient. - Will have significant case volumes to sustain clinical excellence - Be a 24/7 service - Trauma Service have direct responsibility for admitted trauma patients - Will also provide a higher level of trauma related:   - Research   - Multi-disciplinary education   - Data and QI activities   - Prevention and outreach programs   - Leadership role to other hospitals |
| Accreditation Level | **II** |
|  | - Can be either metropolitan or rural based. - Provide comprehensive clinical care for the severely injured to supplement the clinical activities of Level I services. - Clinical aspects should be identical of a Level I service but not necessarily the same level of education/research |
| Accreditation Level | **III** |
|  | - Provide prompt assessment, resuscitation, emergency surgery and stabilization of a small number of seriously injured patients prior to referral and transfer. - Can provide definitive care to a limited number of major trauma patients in concert with Level I trauma service. |
| Accreditation Level | **IV** |
|  | - Capable of providing resuscitation and early stabilization of major trauma patients and prompt referral to higher trauma level. - These levels of services are not intended to care for major trauma patients beyond a safe period for transfer |
