# Supplementary material for: Follow-up after major traumatic injury: a survey of services in Australian and New Zealand public hospitals
Source: BMC Health Serv Res. 2024 May 15;24:630. doi: 10.1186/s12913-024-11105-w (PMC11097478; doi:10.1186/s12913-024-11105-w)
Supplement: Supplementary file 3 — Supplementary Material 3 [file 12913_2024_11105_MOESM3_ESM.docx]

**Additional File 2 – Combined Quality Conceptual Framework – Donabedian and Institute of Medicine (IOM)**

|  | Donabedian | | |
| --- | --- | --- | --- |
| IOM | **STRUCTURE** | **PROCESS** | **OUTCOME** |
| Effectiveness | Available resources  *Economic factors* | Follow up activities | *Goals of follow up: - patient, family organisational*  *Minimum standards* |
| Efficiency | Location to Trauma Centre  *Organisational factors* | Frequency and timing of follow up | Attendance rates  *Economic value* |
| Safety | Qualifications and experience of healthcare staff  Number of staff  MDT v isolated practitioners  Protocols and guidelines | Follow up activities | Identification of ongoing physical/emotional issues  *Standardised reporting – regular reviews*  *Minimum standards* |
| Patient Centeredness | Specialised program v individual services  Delivery methods of trauma follow up | Inclusion and exclusion criteria  Follow up activities.  Patient engagement | Patient satisfaction  *Family satisfaction* |
| Timeliness |  | Frequency and timing of follow-up | *Minimum standards* |
| Equity | Location to Trauma Centre  Specialised program versus individual services | Inclusion and exclusion criteria | *Self-referral* |
| *Italics represent recommended areas based upon gaps identified in scoping review ^(11)^* | | | |
