# Supplementary material for: Follow-up after major traumatic injury: a survey of services in Australian and New Zealand public hospitals
Source: BMC Health Serv Res. 2024 May 15;24:630. doi: 10.1186/s12913-024-11105-w (PMC11097478; doi:10.1186/s12913-024-11105-w)
Supplement: Supplementary file 4 — Supplementary Material 4 [file 12913_2024_11105_MOESM4_ESM.pdf]

Survey - Participant Demographics

Please complete the survey below.

Thank you!

Study Objective
To inform the evaluation of trauma follow-up services, an understanding of the types of trauma follow-up services that are available is first required. Through the data collected in this survey the current landscape of trauma follow-up services will be described and facilitate a greater understanding of what services are available to patients and families.

Overview
Prior to sending the survey to all hospitals involved in the delivery of trauma follow-up care to patients affected by major trauma, we are undertaking content validity testing as an initial first step. This involves review and assessment by expert trauma clinicians, such as yourself, to ensure that each survey item (question) is relevant and has appropriate response options. For each question we would ask you to (i) rate the relevance of the survey item and (ii) comment on the relevance of the response options. There are x questions in total and we anticipate that this survey will take you approximately 45 - 60 mins to complete.

A participant information and consent form is attached to this survey and can be accessed below.

Participant Information and Consent Form

[Attachment: "Study1_PICF_V1.0_15.10.2022_clean.15.01.2023.doc"]

Participant Demographics

Health Discipline	◯ Nurse<br>◯ Doctor<br>◯ Allied Health<br>◯ other - please specify
Health Discipline - Other	____________________
Years of Experience	◯ Up to 1 year<br>◯ Between 2 to 5 years<br>◯ Between 6 to 10 years<br>◯ Over 10 years

Survey - Part 2 - Survey Questions

Each question is presented in two parts.

The first part you are asked to rate the relevancy of the question using a 1 to 4 scale. Please read and rate every question using the scale below.

1 = Not relevant
2 = Somewhat relevant (major changes required)
3 = Quite relevant (minor changes required)
4 = Very relevant

For each survey question that is rated Not Relevant (1), Somewhat Relevant (2) or Quite Relevant (3), we would like you to comment on your responses and why you have chosen that option. This can be documented in the free text comment box associated with the question. For example, Q1 - all hospitals deliver trauma care so designation is not required.

The second part you are asked to make a comment on the relevancy of the responses. Please note that if you rate all the responses relevant, then this box can be left blank.

For the pupose of Content Validity Testing ALL questions are visible to enable you to assess their relevancy; however, in the FINAL version of the survey, branching logic will be used so that subsequent questions which relate to a specific response option are made visible.

At the end of the survey a free text box will be provided for you to add any additional comments OR questions that you think should be included.

Date Completed	______________________________
Hospital Name	______________________________
Patient Population	◯ Adults<br>◯ Paediatrics<br>◯ Both
Country	◯ Australia<br>◯ New Zealand
State/Territory	◯ New South Wales<br>◯ Victoria<br>◯ Queensland<br>◯ South Australia<br>◯ Western Australia<br>◯ Tasmania<br>◯ Northern Territory<br>◯ Australian Capital Territory
Provinces	◯ Auckland<br>◯ New Plymouth<br>◯ Wellington<br>◯ Nelson<br>◯ Canterbury<br>◯ Otago

Structure: the context where care is delivered

Physical Characteristics of the trauma centre/hospital

Question 1

What is the type of hospital at which you work?

Please refer to the attached PDF document for hospital definitions.

◯ Not relevant
◯ Somewhat relevant (major changes required)
◯ Quite relevant (minor changes required)
◯ Very relevant
(Please select one of the above options in terms of relevancy)

Definition of hospital

[Attachment: "Definition of Hospitals.pdf"]

Question 1 - Responses

(i) Trauma Centre

(ii) Tertiary Hospital

(iii) Regional Hospital

(iv) Rural Hospital

Free text comments and suggestions

____
(Please comment on the relevancy of the responses; if all are relevant this can be left blank)

Question 2

What is the current level of trauma centre you work at (as determined by State or Territory or the Royal Australasian College of Surgeons - Australian and Aotearoa New Zealand Trauma Verification Program)

Please refer to the attached PDF document for the level definitions.

◯ Not relevant
◯ Somewhat relevant (major changes required)
◯ Quite relevant (minor changes required)
◯ Very relevant
(Please select one of the above options in terms of relevancy)

Trauma Centre Level

[Attachment: "Trauma Centre Level.pdf"]

Question 2 - Responses

(i) Level I
(ii) Level II
(iii) Level III
(iv) Level IV

____
(Please comment on the relevancy of the responses; if all are relevant this can be left blank)

Free text comments and suggestions

Question 3

Does the Trauma Centre have a 'current' Royal Australasian College of Surgeons, trauma accreditation status?

◯ Not relevant
◯ Somewhat relevant (major changes required)
◯ Quite relevant (minor changes required)
◯ Very relevant
(Please select one of the above options in terms of relevancy)

Question 3 - Responses

(i) Yes
(ii) No
(iii) Unsure

____
(Please comment on the relevancy of the responses; if all are relevant this can be left blank)

Free text comments and suggestions

Question 4

How many funded in-patient beds does your hospital have?

◯ Not relevant
◯ Somewhat relevant (major changes required)
◯ Quite relevant (minor changes required)
◯ Very relevant
(Please select one of the above options in terms of relevancy)

Question 4 - Responses

(i) Up to 49
(ii) Between 50 to 100
(iii) Between 101 to 200
(iv) Between 201 to 300
(v) Between 301 to 400
(vi) Between 401 to 500
(vii) Over 500
(viii) Unsure

____
(Please comment on the relevancy of the responses; if all are relevant this can be left blank)

Free Text Comments and Suggestions

Physical characteristics of the trauma service

Question 5a

Where is the Trauma Follow-Up Service located?

◯ Not relevant
◯ Somewhat relevant (major changes required)
◯ Quite relevant (minor changes required)
◯ Very relevant
(Please select one of the above the options in terms of relevancy)

Question 5a - Responses

(i) At the same hospital as selected above
(ii) At different location separate to the hospital but within the same health service
(iii) At a different location separate to the hospital AND in a different health service
(iv) Not a physical location as service is delivered by telehealth (phone/video)

Free text comments and suggestions

____
(Please comment on the relevancy of the responses; if all relevant this can be left blank)

Question 5b

If the trauma service follow-up is at a different location, please estimate the KM from the hospital to follow-up service

____

Question 6

Does the follow-up service have a designated space?

◯ Not relevant
◯ Somewhat relevant (major changes required)
◯ Quite relevant (minor changes required)
◯ Very relevant
(Please select one of the above options in terms of relevancy)

Question 6 - Responses

(i) Yes, as part of the outpatient department
(ii) Yes, located in a hospital ward
(iii) No designated space
(iv) Other - please specify

Free text comments and suggestions

____
(Please comment on the relvancy of the responses; if all are relevant this can be left blank)

Question 7

Does the follow-up service have adequate disability access i.e. parking, ramps, toilets etc?

◯ Not relevant
◯ Somewhat relevant (major changes required)
◯ Quite relevant (minor changes required)
◯ Very relevant
(Please select one of the above options in terms of relevancy)

Question 7 - Responses

(i) Yes
(ii) No
(iii) Not applicable (telehealth - phone/video)

Free text comments and suggestions

____
(Please comment on the relevancy of the responses; if all are relevant this can be left blank)

Question 8<br><br>Does the follow-up service have a waiting room?	◯ Not relevant<br>◯ Somewhat relevant (major changes required)<br>◯ Quite relevant (minor changes required)<br>◯ Very relevant<br>(Please select one of the above options in terms of relevancy)
Question 8 - Responses<br><br>(i) Yes<br>(ii) No<br>(iii) Not applicable (telehealth - phone/video)<br><br>Free text comments and suggestions	__________________________________<br>(Please comment on the relevancy of the responses; if all are relevant this can be left blank)
Question 9<br><br>Do patients have to pay for parking to attend the follow- up service?	◯ Not relevant<br>◯ Somewhat relevant (major changes required)<br>◯ Quite relevant (minor changes required)<br>◯ Very relevant<br>(Please select one of the above options in terms of relevancy)
Question 9 - Responses<br><br>(i) Yes, but get reimbursed (either fully or partially) by the hospital/follow-up service<br>(ii) Yes, but with no reimbursement<br>(iii) No<br>(iv) Unsure<br><br>Free text comments and suggestions	__________________________________<br>(Please comment on the relevancy of the responses; if all are relevant this can be left blank)
Question 10<br><br>Does the follow-up service, health service or other body (insurance or government) provide transport to attend the follow-up appointment?	◯ Not relevant<br>◯ Somewhat relevant (major changes required)<br>◯ Quite relevant (minor changes required)<br>◯ Very relevant<br>(Please select one of the above options in terms of relevancy)
Question 10 - Responses<br><br>(i) Yes<br>(ii) No<br>(iii) Unsure<br><br>Free text comments and suggestions	__________________________________<br>(Please comment on the relevancy of the responses; if all are relevant this can be left blank)
Question 11<br><br>Please select all transport options that are provided so patients can attend the follow-up appointments?	◯ Not relevant<br>◯ Somewhat relevant (major changes required)<br>◯ Quite relevant (minor changes required)<br>◯ Very relevant<br>(Please select one of the above options in terms of relevancy)

Question 11 - Responses

(i) Taxi vouchers
(ii) Fuel vouchers
(iii) Public transport voucher
(iv) Hospital patient transport
(v) Other - please specify

____
(Please comment on the relevancy of the responses; if all are relevant this can be left blank)

Free Text Suggestions and Comments

Trauma Follow-Up service system infrastructure

Question 12

How is the trauma follow-up service funded?

◯ Not relevant
◯ Somewhat relevant (major changes required)
◯ Quite relevant (minor changes required)
◯ Very relevant
(Please select one of the above options in terms of relevancy)

Question 12 - Responses

(i) Part of trauma service operational budget
(ii) External funding
(iii) Grant funded/hospital foundation/donations
(iv) Funding is not provided on a fixed term
(v) Not funded

Free text comments and suggestions

____
(Please comment on the relevancy of the responses; if all are relevant this can be left blank)

Question 13

Is the funding for the trauma follow-up service permanent?

◯ Not relevant
◯ Somewhat relevant (major changes required)
◯ Quite relevant (minor changes required)
◯ Very relevant

Question 13 - Responses

(i) Yes
(ii) No
(iii) Unsure

Free Text Comments and Suggestions

____
(Please comment on the relevancy of the responses; if all are relevant this can be left blank)

Governance

Question 14

Where does the trauma follow-up service sit in the broader operational structure?

◯ Not relevant
◯ Somewhat relevant (major changes required)
◯ Quite relevant (minor changes required)
◯ Very relevant
(Please select one of the above options in terms of relevancy)

Question 14 - Responses

(i) Within outpatients
(ii) Within the surgical division (orthopaedics, neurosurgery, general surgery, trauma, other)
(iii) Within another division
(iv) Unsure

Free text comments and suggestions

____
(Please comment on the relevancy of the responses; if all are relevant this can be left blank)

Question 15

What is the discipline of the director of the division, where the trauma service follow-up sits within the organisational structure?

◯ Not relevant
◯ Somewhat relevant (major changes required)
◯ Quite relevant (minor changes required)
◯ Very relevant
(Please select one of the above options in terms of relevancy)

Question 15 - Responses

(i) Nursing director
(ii) Medical director
(iii) Allied health director
(iv) Combination (Medical, Nursing, Allied Health)
(v) Administration/Clerical

Free text comments and suggestions

____
(Please comment on the relevancy of the responses; if all are relevant this can be left blank)

Question 16

Does the trauma follow-up service/clinic have a vision or mission statement?

◯ Not relevant
◯ Somewhat relevant (major changes required)
◯ Quite relevant (minor changes required)
◯ Very relevant
(Please select one of the above options in terms of relevancy)

Question 16 - Responses

(i) Yes
(ii) No
(iii) Unsure

Free text comments and suggestions

____
(Please comment on the relevancy of the responses; if all are relevant this can be left blank)

Question 17

Does the trauma follow-up service/clinic have an operational or strategic plan?

◯ Not relevant
◯ Somewhat relevant (major changes required)
◯ Quite relevant (minor changes required)
◯ Very relevant
(Please select one of the above options in terms of relevancy)

Question 17 - Responses

(i) Yes
(ii) No
(iii) Unsure

____
(Please comment on the relevancy of the responses; if all are relevant this can be left blank)

Free text comments and suggestions

Question 18

If the follow-up service has an operational or strategic plan is it:

◯ Not relevant
◯ Somewhat relevant (major changes required)
◯ Quite relevant (minor changes required)
◯ Very relevant
(Please select one of the above options in terms of relevancy)

Question 18 - Responses

(i) Department specific
(ii) Same as the wider health service
(iii) Other - please specify
(iv) - Unsure

____
(Please comment on the relevancy of the responses; if all are relevant this can be left blank)

Free text comments and suggestions

Question 19

How are patients notified of the follow-up appointment? Please select all responses that apply:

◯ Not relevant
◯ Somewhat relevant (major changes required)
◯ Quite relevant (minor changes required)
◯ Very relevant
(Please select one of the above options in terms of relevancy)

Question 19 - Responses

(i) In person
(ii) Phone
(iii) text message
(iv) Email
(v) Letter
(vi) Combination of the above
(vii) Other - please specify

____
(Please comment on the relevancy of the responses; if all are relevant this can be left blank)

Free text comments and suggestions

Question 20

In general, are patients notified of their follow up appointment....

◯ Not relevant
◯ Somewhat relevant (major changes required)
◯ Quite relevant (minor changes required)
◯ Very relevant
(Please select one of the above options in terms of relevancy)

Question 20 - Responses

(i) Before they leave hospital
(ii) After they leave hospital

____
(Please comment on the relevancy of the responses; if all are relevant this can be left blank)

Free text comments and suggestions

Question 21<br><br>If patients are notified of their fllow-up appointment after they leave hospital, is this by....	◯ Not relevant<br>◯ Somewhat relevant (major changes required)<br>◯ Quite relevant (minor changes required)<br>◯ Very relevant<br>(Please select one of the above options in terms of relevancy)
Question 21 - Responses<br><br>(i) Phone<br>(ii) Text message<br>(iii) Email<br>(iv) Letter<br>(v) Telehealth<br>(vi) Combination of the above<br>(vii) Other - please specify<br><br>Free text comments and suggestions	__________________________________<br>(Please comment on the relevancy of the responses; if all are relevant this can be left blank)
Question 22<br><br>Are family members or close others routinely encouraged to attend the follow-up appointments?	__________________________________<br>(Please select one of the above options in terms of relevancy)
Question 22 - Responses<br><br>(i) Yes, if they are with the patient when discussion of the appointment occurs<br>(ii) Yes, they are contacted in addition to the patient<br>(iii) No<br><br>Free text comments and suggestions	__________________________________<br>(Please comment on the relevancy of the responses; if all are relevant this can be left blank)
Question 23<br><br>In general, are patients informed of the reason(s) for the follow-up appointment(s); for example wound care, further investigations (bloods imaging):	◯ Not relevant<br>◯ Somewhat relevant (major changes required)<br>◯ Quite relevant (minor changes required)<br>◯ Very relevant<br>(Please select one of the above options in terms of relevancy)
Question 23 - Responses<br><br>(i) Yes, if the patient is informed of the appointment in person<br>(ii) Yes, it is incoporated into the letter, text, email etc<br>(iii) Yes, but the reason is standard i.e. 'you are required to attend for a 'check-up' etc<br>(iv) No<br><br>Free text comments and suggestions	__________________________________<br>(Please comment on the relevancy of the responses; if all are relevant this can be left blank)
Question 24<br><br>How is the follow-up service delivered? Please select all that apply:	◯ Not relevant<br>◯ Somewhat relevant (major changes required)<br>◯ Quite relevant (minor changes required)<br>◯ Very relevant<br>(Please select one of the above options in terms of relevancy)

Question 24 - Responses

(i) in person
(ii) Telehealth (video/phone)
(iii) Combination of the above
(iv) Other - please specify

Free text comments and suggestions

____
(Please comment on the relevancy of the responses; if all are relevant this can be left blank)

Question 25

Does the follow-up service have protocols or guidelines which outline which delivery method is used and why?

◯ Not relevant
◯ Somewhat relevant (major changes required)
◯ Quite relevant (minor changes required)
◯ Very relevant
(Please select one of the above options in terms of relevancy)

Question 25 - Responses

(i) Yes - please specify
(ii) No

Free text comments and suggestions

____
(Please comment on the relevancy of the responses; if all are relevant this can be left blank)

Question 26

Are patients offered a choice of delivery methods for the follow-up service:

◯ Not relevant
◯ Somewhat relevant (major changes required)
◯ Quite relevant (minor changes required)
◯ Very relevant
(Please select one of the above options in terms of relevancy)

Question 26 - Responses

(i) Yes, all patients are offered a choice
(ii) Yes, but only if patients live outside the local catchment area
(iii) Yes, but it is dependent on the reason for the follow-up appointment i.e. wound care is in person only
(iv) No
(v) Unsure

Free text comments and suggestions

____
(Please comment on the relevancy of the responses; if all are relevant this can be left blank)

Question 27

Did the follow-up service delivery method change with COVID-19? Please select all that apply

◯ Not relevant
◯ Somewhat relevant (major changes required)
◯ Quite relevant (minor changes required)
◯ Very relevant
(Please select one of the above options in terms of relevancy)

Question 27 - Responses

(i) Yes, the follow-up service was ceased during lockdown periods
(ii) Yes, the follow-up service was ceased completely
(iii) Yes, the follow-up service changed to telephone/telehealth only
(iv) Yes, the follow-up service was provided by GPs
(v) Yes, the inclusion/exclusion to identify patients who should attend the follow-up service was altered
(vi) Other - please specify
(vii) No
(vii) Unsure

Free text comments and suggestions

____
(Please comment on the relevancy of the responses; if all are relevant this can be left blank)

Question 28

If the follow-up service uses telehealth, is it:

◯ Not relevant
◯ Somewhat relevant (major changes required)
◯ Quite relevant (minor changes required)
◯ Very relevant
(Please select one of the above options in terms of relevancy)

Question 28 - Responses

(i) Phone only
(ii) Video only
(iii) Combination of phone and video
(iv) Other - please specify

Free text comments and suggestions

____
(Please comment on the relevancy of the responses; if all are relevant this can be left blank)

Question 29

If the follow-up service uses telehealth, is this via:

◯ Not relevant
◯ Somewhat relevant (major changes required)
◯ Quite relevant (minor changes required)
◯ Very relevant
(Please select one of the above options in terms of relevancy)

Question 29 - Responses

(i) Microsoft TEAMS
(ii) ZOOM
(iii) Skype
(iv) Facetime
(v) Hospital specific software
(vi) Other - please specify

Free text comments and suggestions

____
(Please comment on the relevancy of the responses; if all are relevant this can be left blank)

Question 30

Is telehealth direct to the patient?

◯ Not relevant
◯ Somewhat relevant (major changes required)
◯ Quite relevant (minor changes required)
◯ Very relevant
(Please select one of the above options in terms of relevancy)

Question 30 - Responses

(i) Yes, they can access the telehealth on their own electronic device
(ii) No, patients have to attend a local hospital/GP to access telehealth
(iii) No, other - please specify

____
(Please comment on the relevancy of the responses; if all are relevant this can be left blank)

Free text comments and suggestions

Question 31

If telehealth is used, where is the health professional located?

◯ Not relevant
◯ Somewhat relevant (major changes required)
◯ Quite relevant (minor changes required)
◯ Very relevant
(Please select one of the above options in terms of relevancy)

Question 31 - Responses

(i) In a room occupied only by the trauma follow-up care health professional(s)
(ii) In a shared space with other health professionals not involved in the trauma follow-up care

____
(Please comment on the relevancy of the responses; if all are relevant this can be left blank)

Free text comments and suggestions

Equipment

Question 32

Please identify all equipment/resources available to the staff in the follow-up service:

◯ Not relevant
◯ Somewhat relevant (major changes required)
◯ Quite relevant (minor changes required)
◯ Very relevant
(Please select one of the above options in terms of relevancy)

Question 32 - Responses

(i) Electronic Medical Records
(ii) Access to imaging reports (Xray, CT, MRI, USS)
(iii) Access to pathology results
(iv) Access to reports (imaging, pathology) obtained externally to the hospital
(v) Telehealth - video equipment
(vi) Physical examination: stethescope, otoscope
(vii) Wound care: dressings, sutures etc
(vii) Pathology services
(viii) Other - please specify

Free text comments and suggestions

____
(Please comment on the relevancy of the responses; if all are relevant this can be left blank)

Human Resources

Question 33

Would you consider the trauma follow-up service to be predominantly:

- ◯ Not relevant
- ◯ Somewhat relevant (major changes required)
- ◯ Quite relevant (minor changes required)
- ◯ Very relevant

(Please select one of the above options in terms of relevancy)

Question 33 - Responses

(i) Nurse led
(ii) Doctor led
(iii) Allied Health led
(iv) Multi-discplinary team led

Free text comments and suggestions

____
(Please comment on the relevancy of the responses; if all are relevant this can be left blank)

Question 34

Who are the staff that regularly work in the trauma follow-up service? Please select all that apply:

- ◯ Not relevant
- ◯ Somewhat relevant (major changes required)
- ◯ Quite relevant (minor changes required)
- ◯ Very relevant

(Please select one of the above options in terms of relevancy)

Question 34 - Responses

(i) Nurse
(ii) Surgeon
(iii) Geriatrician
(iv) Physiotherapist
(v) Occupational Therapist
(vi) Speech Pathology
(vii) Dietician
(viii) Rehabilitation Service
(ix) Soical work
(x) Mental Health Services - Psychiatrist, Mental Health Nurses
(xi) Psychologist
(xii) Neuropsychologist
(xiii) Pain management services
(xiv) Orthotics and/or prosthetics
(xv) Aboriginal and Torres Strait Islander Services
(xvi) Interpreter services
(xvii) Administration/Clerical services
(xviii) Financial support (advice only)
(xix) Legal support
(xx) Other - please specify

Free text comments and suggestions

____
(Please comment on the relevancy of the responses; if all are relevant this can be left blank)

Question 35

Do staff receive training (informal or formal) training to work in the follow-up service?

- ◯ Not relevant
- ◯ Somewhat relevant (major changes required)
- ◯ Quite relevant (minor changes required)
- ◯ Very relevant

(Please select one of the above options in terms of relevancy)

Question 35 - Responses<br><br>(i) Yes - please specify<br>(ii) No<br>(iii) Unsure<br><br>Free text comments and suggestions	__________________________________<br>(Please comment on the relevancy of the responses; if all are relevant this can be left blank)
Question 36<br><br>Does the follow-up service have permanent staff to deliver the service ensuring a continuous service (i.e., not cancelled in times of staff sickness or annual leave)?	◯ Not relevant<br>◯ Somewhat relevant (major changes required)<br>◯ Quite relevant (minor changes required)<br>◯ Very relevant<br>(Please select one of the above options in terms of relevancy)
Question 36 - responses<br><br>(i) Yes<br>(ii) No<br>(iii) Unsure<br><br>Free text comments and suggestions	__________________________________<br>(Please comment on the relevancy of the responses; if all are relevant this can be left blank)
Question 37<br><br>Do the patients attending the follow-up service regularly see the same health care staff member or team?	◯ Not relevant<br>◯ Somewhat relevant (major changes required)<br>◯ Quite relevant (minor changes required)<br>◯ Very relevant<br>(Please select one of the above options in terms of relevancy)
Question 37 - Responses<br><br>(i) Yes, they see the same staff member/team<br>(ii) No, they see different staff members<br>(iii) It depends upon the needs of the patient i.e., for ongoing pain they see a pain specialist<br><br>Free text comments and suggestions	__________________________________<br>(Please comment on the relevancy of the responses; if all are relevant this can be left blank)
Question 38<br><br>Does the follow-up service have regular or consistent access to healthcare professionals from the following discplines/services? Please select all that appy:	◯ Not relevant<br>◯ Somewhat relevant (major changes required)<br>◯ Quite relevant (minor changes required)<br>◯ Very relevant<br>(Please select one of the above options in terms of relevancy)

Question 38 - Responses

(i) Nurse
(ii) Surgeon
(iii) Geriatrician
(iv) Physiotherapist
(v) Occupational Therapist
(vi) Speech Pathology
(vii) Dietician
(viii) Rehabilitation Service
(ix) Soical work
(x) Mental Health Services - Psychiatrist, Mental Health Nurses
(xi) Psychologist
(xii) Neuropsychologist
(xiii) Pain management services
(xiv) Orthotics and/or prosthetics
(xv) Aboriginal and Torres Strait Islander Services
(xvi) Interpreter services
(xvii) Administration/Clerical services
(xviii) Financial support (advice only)
(xix) Legal support
(xx) Other - please specify

Free text comments and suggestions

____
(Please comment on the relevancy of the responses; if all are relevant this can be left blank)

Process - the combination of the actions that make up the follow-up service delivery

Service Delivery - who

Question 39<br><br>Is the follow-up service part of or in conjunction with a specific follow-up program such as the Trauma Survivor Network?	◯ Not relevant<br>◯ Somewhat relevant (major changes required)<br>◯ Quite relevant (minor changes required)<br>◯ Very relevant<br>(Please select one of the above options in terms of relevancy)
Question 39 - Responses<br><br>(i) No, the follow-up service is a stand alone, locally derived service<br>(ii) Yes, the follow-up service includes specific follow-up programs (for eg. Trauma Survivors Network)<br>(iii) Other - please specify<br><br>Free text comments and suggestions	____<br>(Please comment on the relevancy of the responses; if all are relevant this can be left blank)

Inclusion and Exclusion Criteria

Question 40

How are trauma patients selected to attend the follow-up service? Please select all that appy:

◯ Not relevant
◯ Somewhat relevant (major changes required)
◯ Quite relevant (minor changes required)
◯ Very relevant
(Please select one of the above options in terms of relevancy)

Question 40 - Responses

(i) All trauma patients are eligible to attend
(ii) Major trauma patients only (Injury Severity Score > 12)
(iii) Specific inclusion criteria
(iv) Part of an injury or treatment pathway (such as blunt chest trauma)
(v) GP referral
(vi) Identified by other team members from the wider health organisation
(vii) Self-referral by the patient
(viii) Referral by a family member/friend/informal carer
(ix) Dependent upon the proximity to the follow-up service/hospital catchment area
Free text comments and suggestions

____
(Please comment on the relevancy of the responses; if all are relevant this can be left blank)

Question 41

Does the trauma follow-up service incorporate services for families or close others of trauma patients?

◯ Not relevant
◯ Somewhat relevant (major changes required)
◯ Quite relevant (minor changes required)
◯ Very relevant
(Please select one of the above options in terms of relevancy)

Question 41 - Responses

(i) Yes - please specify
(ii) No
(iii) Unsure

Free text comments or suggestions

____
(Please comment on the relevancy of the responses; if all are relevant this can be left blank)

Service Delivery - why

Question 42<br><br>Why are patients asked to attend a follow-up service? Please select all that apply:	◯ Not relevant<br>◯ Somewhat relevant (major changes required)<br>◯ Quite relevant (minor changes required)<br>◯ Very relevant<br>(Please select one of the above options in terms of relevancy)
Question 42 - Responses<br><br>(i) Routine or 'check-up' care<br>(ii) Specific ongoing care requirement<br>(iii) Emotional or psychological support<br>(iv) Part of protocolised care<br>(v) Other - please specify<br><br>Free text comments and suggestions	____<br>(Please comment on the relevancy of the responses; if all are relevant this can be left blank)
Question 43<br><br>Are patients offered more than one trauma follow-up appointment?	◯ Not relevant<br>◯ Somewhat relevant (major changes required)<br>◯ Quite relevant (minor changes required)<br>◯ Very relevant<br>(Please select one of the above options in terms of relevancy)
Question 43 - Responses<br><br>(i) Yes<br>(ii) Yes, but it depends on certain criteria<br>(iii) No<br><br>Free text comments and suggestions	____<br>(Please comment on the relevancy of the responses; if all are relevant this can be left blank)
Question 44<br><br>For patients who require multiple appointments, what is the general reason?	◯ Not relevant<br>◯ Somewhat relevant (major changes required)<br>◯ Quite relevant (minor changes required)<br>◯ Very relevant<br>(Please select one of the above options in terms of relevancy)
Question 44 - Responses<br><br>(i) Ongoing clinical (physical) requirements<br>(ii) Ongoing emotional requirements<br>(iii) Combination of both clinical and emotional requirements<br>(iv) At the patients request<br>(v) At the family's/friend/informal carer request<br>(vi) At the request of primary care (GP etc)<br><br>Free text comments and suggestions	____<br>(Please comment on the relevancy of the responses; if all are relevant this can be left blank)

Service Delivery - when

Question 45

What time points are patients invited for their first appointment? Please select all that apply:

◯ Not relevant
◯ Somewhat relevant (major changes required)
◯ Quite relevant (minor changes required)
◯ Very relevant
(Please select one of the above options in terms of relevancy)

Question 45 - Responses

(i) Within 2 weeks of discharge from hospital
(ii) Within 3-4 weeks of discharge from hospital
(iii) Within 5-6 weeks of discharge from hospital
(iv) Within 7-8 weeks of discharge from hospital
(v) More than 8 weeks after discsharge from hospital
(vi) Other - please specify

Free text comments and suggestions

____
(Please comment on the relevancy of the responses; if all are relevant this can be left blank)

Question 46

Does the follow-up service have a discharge criteria?

◯ Not relevant
◯ Somewhat relevant (major changes required)
◯ Quite relevant (minor changes required)
◯ Very relevant
(Please select one of the above options in terms of relevancy)

Question 46 - Responses

(i) Yes
(ii) No
(iii) Unsure

Free text comments and suggestions

____
(Please comment on the relevancy of the responses; if all are relevant this can be left blank)

Service Delivery - frequency

Question 47

How frequently does the follow-up service run?

◯ Not relevant
◯ Somewhat relevant (major changes required)
◯ Quite relevant (minor changes required)
◯ Very relevant
(Please select one of the above options in terms of relevancy)

Question 47 - Responses

(i) Daily (Monday to Friday only)
(ii) 7 days per week
(iii) 3-4 times per week
(iv) 1-2 times per week
(v) Weekly
(vi) Fortnightly

____
(Please comment on the relevancy of the responses; if all are relevant this can be left blank)

Free text comments and suggestions

Service Delivery - Follow-up activities

Question 48

What activities occur during a follow-up appointment?
Please select all that apply:

◯ Not relevant
◯ Somewhat relevant (major changes required)
◯ Quite relevant (minor changes required)
◯ Very relevant
(Please select one of the above options in terms of relevancy)

Question 48 - Responses

(i) Physical examination/assessment
(ii) Pain Assessment
(iii) Quality of life assessment
(iv) Mental Health Assessment (PTSD/Depression tool)
(v) Medication review
(vi) Imaging review
(vii) Pathology review
(viii) Further investigations ordered - imaging/pathology/other
(ix) Education of patients
(x) Education of family members or close others
(xi) Emotional support
(xii) Discussion about recovery journey
(xiii) Family assessment
(xiv) Repeat appointments scheduled
(xv) Referrals for additional services
(xvi) Other - please specify
Free text comments and suggestions

____
(Please comment on the relevancy of the responses; if all are relevant this can be left blank)

Protocols, Guidelines and Referral Pathways

Question 49

Does the follow-up service use specific protocols/guidelines/procedures/referral pathways?

◯ Not relevant
◯ Somewhat relevant (major changes required)
◯ Quite relevant (minor changes required)
◯ Very relevant
(Please select one of the above options in terms of relevancy)

Question 49 - Responses

(i) Yes
(ii) No
(iii) Unsure

Free text comments and suggestions

____
(Please comment on the relevancy of the responses; if all are relevant this can be left blank)

Question 50

Please identify the protocols, guidelines and/or procedures that are available in your follow-up service?

◯ Not relevant
◯ Somewhat relevant (major changes required)
◯ Quite relevant (minor changes required)
◯ Very relevant
(Please select one of the above options in terms of relevancy)

Question 50 - Responses

(i) Pain Management
(ii) Injury specific (Blunt Chest Trauma, Splenic Injury)
(iii) Psychological and emotional health
(iv) Readmission to hospital
(v) Other - plese specify

Free text comments and suggestions

____
(Please comment on the relevancy of the responses; if all are relevant this can be left blank)

Question 51

Does the follow-up service have specific referral pathways?

◯ Not relevant
◯ Somewhat relevant (major changes required)
◯ Quite relevant (minor changes required)
◯ Very relevant
(Please select one of the above options in terms of relevancy)

Question 51 - Responses

(i) Yes
(ii) No
(iii) Unsure

Free text comments and suggestions

____
(Please comment on the relevancy of the responses; if all are relevant this can be left blank)

Question 52

Please identify the referral pathways available; please select all that apply:

◯ Not relevant
◯ Somewhat relevant (major changes required)
◯ Quite relevant (minor changes required)
◯ Very relevant
(Please select one of the above options in terms of relevancy)

Question 52 - Responses

(i) Nurse (general or specialist)
(ii) Surgeon
(iii) Geriatrician
(iv) Physiotherapist
(v) Occupational Therapist
(vi) Speech Pathology
(vii) Dietician
(viii) Rehabilitation Service
(ix) Soical work
(x) Mental Health Services - Psychiatrist, Mental Health Nurses
(xi) Psychologist
(xii) Neuropsychologist
(xiii) Pain management services
(xiv) Orthotics and/or prosthetics
(xv) Aboriginal and Torres Strait Islander Services
(xvi) Interpreter services
(xvii) Administration/Clerical services
(xviii) Financial support (advice only)
(xix) Legal support
(xx) Other - please specify

Free text comments and suggestions

____
(Please comment on the relevancy of the responses; if all are relevant this can be left blank)

Question 53

Where are these services located?

◯ Not relevant
◯ Somewhat relevant (major changes required)
◯ Quite relevant (minor changes required)
◯ Very relevant
(Please select one of the above options in terms of relevancy)

Question 53 - Responses

(i) In the hospital where the follow-up service is located
(ii) Other public hospital (same health service)
(iii) Other public hospital (different health service)
(iv) Private hospital
(v) Primary Care

Free text comments and suggestions

____
(Please comment on the relevancy of the responses; if all are relevant this can be left blank)

Question 54

Does the follow-up service provide educational or health promotional resources (i.e., printed or online) to patients?

◯ Not relevant
◯ Somewhat relevant (major changes required)
◯ Quite relevant (minor changes required)
◯ Very relevant
(Please select one of the above options in terms of relevancy)

Question 54 - Responses

(i) Yes
(ii) No
(iii) Unsure

Free text comments and suggestions

____
(Please comment on the relevancy of the responses; if all are relevant this can be left blank)

Question 55<br><br>Please select all education and/or health promotion materials that are available:	◯ Not relevant<br>◯ Somewhat relevant (major changes required)<br>◯ Quite relevant (minor changes required)<br>◯ Very relevant<br>(Please select one of the above options in terms of relevancy)
Question 55 - Responses<br><br>(i) Pain<br>(ii) Blunt Chest trauma/Chest Injuries<br>(iii) Pelvic Injuries<br>(iv) Splenic/Liver injuries<br>(v) Orthopaedic Injuries - upper limbs<br>(vi) Orthopaedic Injuries (lower limbs)<br>(vii) Traumatic Brain Injuries<br>(viii) Emotional Health (mental Health/Social Support)<br>(ix) Alcohol and drug<br>(x) Financial support (advice only)<br>(xi) Legal support<br>(xii) Other - please specify<br><br>Free text comments and suggestions	__________________________________<br>(Please comment on the relevancy of the responses; if all are relevant this can be left blank)
Question 56<br><br>Does the follow-up service provide educational or health promotional resources (i.e., printed or online) to families or close others?	◯ Not relevant<br>◯ Somewhat relevant (major changes required)<br>◯ Quite relevant (minor changes required)<br>◯ Very relevant<br>(Please select one of the above options in terms of relevancy)
Question 56 - Responses<br><br>(i) Yes<br>(ii) No<br>(iii) Unsure<br><br>Free text comments and suggestions	__________________________________<br>(Please comment on the relevancy of the responses; if all are relevant this can be left blank)
Question 57<br><br>Please select all education and/or health promotion materials that are available for families or close others:	◯ Not relevant<br>◯ Somewhat relevant (major changes required)<br>◯ Quite relevant (minor changes required)<br>◯ Very relevant<br>(Please select one of the above options in terms of relevancy)

Question 57 - Responses

(i) Pain
(ii) Blunt Chest trauma/Chest Injuries
(iii) Pelvic Injuries
(iv) Splenic/Liver injuries
(v) Orthopaedic Injuries - upper limbs
(vi) Orthopaedic Injuries (lower limbs)
(vii) Traumatic Brain Injuries
(viii) Emotional Health (mental Health/Social Support)
(ix) Alcohol and drug
(x) Financial support (advice only)
(xi) Legal support
(xii) Other - please specify

Free text comments and suggestions

____
(Please comment on the relevancy of the responses; if all are relevant this can be left blank)

Question 58

Does the follow-up service use specific assessment tools with patients?

◯ Not relevant
◯ Somewhat relevant (major changes required)
◯ Quite relevant (minor changes required)
◯ Very relevant
(Please select one of the above options in terms of relevancy)

Question 58 - Responses

(i) Yes
(ii) No
(iii) Unsure

Free text comments and suggestions

____
(Please comment on the relevancy of the responses; if all are relevant this can be left blank)

Question 59

Please identify all assessment tools used:

◯ Not relevant
◯ Somewhat relevant (major changes required)
◯ Quite relevant (minor changes required)
◯ Very relevant
(Please select one of the above options in terms of relevancy)

Question 59 - Responses

(i) Quality of Life (SF-12/36, EQ-5D)
(ii) Pain assessment tool (Numerical Rating Scales, Brief Pain Inventory)
(iii) Post-traumatic Stress Disorder (Screening)
(iv) Post-traumatic Stress Disorder (Diagnosis)
(v) Depression (screening)
(vi) Depression (Diagnosis)
(vii) Anxiety
(viii) Mental Health
(ix) Alcohol use
(x) Substance use
(xi) Return to work
(xii) Cognitive functioning
(xiii) Family assessment
(xiv) Other - please specify

Free text comments and suggestions

____
(Please comment on the relevancy of the responses; if all are relevant this can be left blank)

Question 60<br><br>Does the follow-up service use specific assessment tools with families or close others?	◯ Not relevant<br>◯ Somewhat relevant (major changes required)<br>◯ Quite relevant (minor changes required)<br>◯ Very relevant<br>(Please select one of the above options in terms of relevancy)
Question 60 - Responses<br><br>(i) Yes<br>(ii) No<br>(iii) Unsure<br><br>Free text comments and suggestions	__________________________________<br>(Please comment on the relevancy of the responses; if all are relevant this can be left blank)
Question 61<br><br>Please identify all the assessment tools used with families or close others:	◯ Not relevant<br>◯ Somewhat relevant (major changes required)<br>◯ Quite relevant (minor changes required)<br>◯ Very relevant<br>(Please select one of the above options in terms of relevancy)
Question 61 - Responses<br><br>(i) Quality of Life (SF-12/36, EQ-5D)<br>(ii) Pain assessment tool (Numerical Rating Scales, Brief Pain Inventory)<br>(iii) Post-traumatic Stress Disorder (Screening)<br>(iv) Post-traumatic Stress Disorder (Diagnosis)<br>(v) Depression (screening)<br>(vi) Depression (Diagnosis)<br>(vii) Anxiety<br>(viii) Mental Health<br>(ix) Alcohol use<br>(x) Substance use<br>(xi) Return to work<br>(xii) Cognitive functioning<br>(xiii) Family assessment<br>(xiv) Other - please specify<br><br>Free text comments and suggestions	__________________________________<br>(Please comment on the relevancy of the responses; if all are relevant this can be left blank)

Outcomes - the effects of health care

Evaluation and Satisfaction

Question 62

Does the service evaluate patient satisfaction with the follow-up care?

- ◯ Not relevant
- ◯ Somewhat relevant (major changes required)
- ◯ Quite relevant (minor changes required)
- ◯ Very relevant

(Please select one of the above options in terms of relevancy)

Question 62 - Responses

(i) Yes, every patient is asked to complete a satisfaction survey
(ii) Yes, as part of wider hospital or health service surveys
(iii) Yes, occassionally, but not routinely
(iv) No
(v) Unsure

Free text comments and suggestions

____
(Please comment on the relevancy of the responses; if all are relevant this can be left blank)

Question 63

How frequently is the data from the satisfaction evaluations reviewed?

- ◯ Not relevant
- ◯ Somewhat relevant (major changes required)
- ◯ Quite relevant (minor changes required)
- ◯ Very relevant

(Please select one of the above options in terms of relevancy)

Question 63 - Responses

(i) Weekly
(ii) Monthly
(iii) Every 3 months
(iv) Every 6 months
(v) Annually
(vi) Other - please specify
(vii) Unsure

Free text comments and suggestions

____
(Please comment on the relevancy of the responses; if all are relevant this can be left blank)

Question 64

Does the service regularly evaluate family satisfaction with the follow-up care?

- ◯ Not relevant
- ◯ Somewhat relevant (major changes required)
- ◯ Quite relevant (minor changes required)
- ◯ Very relevant

(Please select one of the above options in terms of relevancy)

Question 64 - Responses

(i) Yes, all family members are asked to complete a satisfaction survey
(ii) Yes, occassionally but not routinely
(iii) Yes, as part of wider hospital surveys
(iv) No
(v) Unsure

Free text comments and suggestions

____
(Please comment on the relevancy of the responses; if all are relevant this can be left blank)

Question 65

Does the follow- up service regularly evaluate staff experience with the follow-up care?

◯ Not relevant
◯ Somewhat relevant (major changes required)
◯ Quite relevant (minor changes required)
◯ Very relevant
(Please select one of the above options in terms of relevancy)

Question 65 - Responses

(i) Yes, all trauma follow-up staff members are asked to complete a staff experience survey
(ii) Yes, occasionally but not routinely
(ii) Yes, as part of the wider hospital health service
(iv) No
(v) Unsure

Free text comments and suggestions

____
(Please comment on the relevancy of the responses; if all are relevant this can be left blank)

Quality

Question 66<br><br>Does the follow-up service have specific Key Performance Indictators such as attendance rates or hospital admission from clinic?	◯ Not relevant<br>◯ Somewhat relevant (major changes required)<br>◯ Quite relevant (minor changes required)<br>◯ Very relevant<br>(Please select one of the above options in terms of relevancy)
Question 66 - Responses<br><br>(i) Yes - please specify<br>(ii) No<br>(iii) Unsure<br><br>Free text comments and suggestions	__________________________________<br>(Please comment on the relevancy of the responses; if all are relevant this can be left blank)
Question 67<br><br>Does the follow-up service have a procedure to deal with complaints from patients?	◯ Not relevant<br>◯ Somewhat relevant (major changes required)<br>◯ Quite relevant (minor changes required)<br>◯ Very relevant<br>(Please select one of the above options in terms of relevancy)
Q67 - Responses<br><br>(i) Yes<br>(ii) No<br>(iii) Unsure<br><br>Free text comments and suggestions	__________________________________<br>(Please comment on the relevancy of the responses; if all are relevant this can be left blank)
Question 68<br><br>Does the follow-up service have a procedure to deal with complaints from family members and/or informal carers?	◯ Not relevant<br>◯ Somewhat relevant (major changes required)<br>◯ Quite relevant (minor changes required)<br>◯ Very relevant<br>(Please select one of the above options in terms of relevancy)
Q68 - Responses<br><br>(i) Yes<br>(ii) No<br>(iii) Unsure<br><br>Free text comments and suggestions	__________________________________<br>(Please comment on the relevancy of the responses; if all are relevant this can be left blank)
Question 69<br><br>Does the follow-up service have a procedure to deal with incident reporting?	◯ Not relevant<br>◯ Somewhat relevant (major changes required)<br>◯ Quite relevant (minor changes required)<br>◯ Very relevant<br>(Please select one of the above options in terms of relevancy)

Q69 - Responses

(i) Yes
(ii)
No
(iii) Unsure

Free text comments and suggestions

(Please comment on the relevancy of the responses; if all are relevant this can be left blank)

Question 70

Please leave any additional comments or questions that you believe should be included.

Study 2 - Clinician Interviews

Please complete the survey below.

Thank you!

Study 2 - Trauma Clinician Interviews

The second study in this PhD is to examine and explore the experiences of trauma clinicians in providing a trauma follow-up service to patients affected by major trauma.

Using semi-structured interviews, understanding what works well and why, and which aspects of follow-up care are evaluated and how the quality of follow-up care is evaluated will help to provide a deeper understanding of trauma follow-up care.

If you would like to register your interest in being considered for an interview either:

(1) Click on 'Watch video' below and complete the form

Or

(2) Please email your name, contact details (email/phone), name of hospital and country/state/territory or province to:
Elizabeth Wake (PhD Candidate)
liz.wake@griffithuni.edu.au
