# Supplementary material for: Follow-up after major traumatic injury: a survey of services in Australian and New Zealand public hospitals
Source: BMC Health Serv Res. 2024 May 15;24:630. doi: 10.1186/s12913-024-11105-w (PMC11097478; doi:10.1186/s12913-024-11105-w)
Supplement: Supplementary file 5 — Supplementary Material 5 [file 12913_2024_11105_MOESM5_ESM.docx]

**Additional File 4 – Content Validation – Results**

Table 1 - Content Validity – Participant Demographics

Participant Demographics

|  | Responded  N = 7 | Invited  N = 16 |
| --- | --- | --- |
| Response Rate | 43.8% |  |
| Discipline   - Nurse - Doctor - Allied Health | 3 (42.9%)  3 (42.9%)  1 (14.2%) | 8 (50%)  7 (44%)  1 (6%) |
| Experience   - 6 to 10 years - Over 10 years | 1 (12.4%)  6 (85.7%) | -  - |
| Country   - Australia - New Zealand | 6 (85.7%)  1 (14.3%) | 14 (87.5%)  2 (12.5%) |
| Patient Population   - Adults - Paediatrics - Both | 5 (71.4%)  -  2 (28.6%) | -  -  - |

States, Territories or Provinces represented:

- Australia: Victoria, Western Australia, Australian Capital Territory, New South Wales
- New Zealand: Auckland

**Content Validity Index – Results**

Based on written feedback and following discussion with the research team, the structure of the final survey was modified to facilitate ease of use. Survey items were also combined to reduce the overall length of the survey.

| **Structure – the context where care is delivered** | | | | | |
| --- | --- | --- | --- | --- | --- |
| **Item No.** | **No. of Experts in Agreement** | **Item CVI** | **Free text comments** | **Decision** | **Action(s)** |
| 1 | 7 | 1.00 | Confusing; All hospitals that take major trauma can validly call themselves TC. Some are tertiary and some are regional.  I am not understanding the question. We are a level 1 major adult trauma service as well as a tertiary hospital so fall into two responses. | **Retained** | Based on written feedback, Items 1,2 and 3 were revised to ensure clarity of the questions. |
| 2 | 7 | 1.00 | This question is slightly confusing in relation to Q1. I interpreted Q1 as determined by state. Verification allows understanding of expected resources, but does not account for mortality rates, LOS, HAC rates, therefore provides little more information than Q1. | **Retained** |  |
| 3 | 6 | 0.86 | This question is slightly confusing in relation to Q1. | **Retained** |  |
| 4 | 6 | 0.86 | Funded? Are there unfunded beds in some places? | **Retained** | Discussion with research team the term ‘funded’ was removed, but the item remains. |
| 5 | 7 | 1.00 | Surely this depends on the injuries and the location of domicile of the injured patient. In XX about 20% of patients are injured in a location other than their domiciled region. Also, if the patient is transferred to a tertiary centre the FU is usually in the regional hospital unless the condition is one only managed in the tertiary hospital. The TBI might be followed up in the tertiary hospital but the ortho injuries might be followed up at the regional hospital.  Where there may be challenges for participants is the definition of trauma follow-up and that for many if not most, follow-up is split between the trauma service and subspeciality clinics. | **Retained** | Discussion with the research team regarding ‘trauma service follow-up’ and ‘follow-up’ of trauma patients based on the written feedback. Item 5 was revised and developed to ensure both categories outlined above were included.  These revisions also forced further revision of the structure of the survey: Items that were originally in the process section (relating to the provision of staff) were relocated to the structure section. |
| 6 | 5 | 0.71 | Is it the space or is it having access to a clinic? You may find that some hospitals utilise telehealth rather than face to face, and again are you confining this to a defined trauma service clinic that performed an array of data collection long term outcome metrics, or are you asking about any clinic? Potential for a large volume of free text responses. | **Retained** | Discussion with the research team regarding the written feedback and the Item CVI being rated below 0.78. To ensure that both face to face and telehealth was captured, this item remained, however, additional items in relation to telehealth were added. |
| 7 | 5 | 0.71 | If the purpose is to understand accessibility, I would think that all health services are bound by the disability act and access is part of accreditation standards. See Australian Health facility guidelines. | **Removed** | Based on the written feedback and the CVI item score this item was removed. |
| 8 | 5 | 0.71 | As above, AusHFG require this – unless your purpose is to audit compliance. | **Retained** | Discussion with the research team to retain this item, despite the CVI score; this is to ensure equality between face to face and telehealth questions. |
| 9 | 6 | 0.86 |  | **Retained** |  |
| 10 | 5 | 0.71 |  | **Retained** | Discussion with the research team regarding the Item CVI being rated below 0.78. Decision made to retain as literature suggests accessibility to follow-up appointment is a factor on compliance. |
| 11 | 5 | 0.71 |  | **Retained** |  |
| 12 | 6 | 0.86 | Funding may be split which may challenge participants. I would suggest that for many, budget and funding is not necessarily visible or understood. | **Retained** |  |
| 13 | 7 | 1.00 |  | **Retained** |  |
| 14 | 7 | 1.00 | See previous responses regarding funding visibility. Outpatient funding is often split with nursing and material resources within ambulatory budget and medical funding as part of the divisional envelope. This means governance can be nuanced. | **Retained** |  |
| 15 | 7 | 1.00 | This doesn’t include surgical vs physician and should.  Definitions here required. Are you asking for clinical director, unit director or executive director? Different states use different structures. Each of these roles within the division have a governance role in the activity and oversight of an outpatient service. | **Removed** | Discussion with the research team regarding the written feedback. Due to high level of variation expected, decision was made to remove item. |
| 16 | 5 | 0.71 |  | **Retained** | Written feedback identifies the relevancy of item. Discussion with the research team as Item CVI being rated below 0.78. Decision to retain items as they provide data which can be related to the evaluation of follow-up services. |
| 17 | 5 | 0.71 |  | **Retained** |  |
| 18 | 4 | 0.57 | Responses suitable. | **Retained** |  |
| 19 | 7 | 1.00 |  | **Retained** |  |
| 20 | 6 | 0.86 |  | **Retained** |  |
| 21 | 7 | 1.00 |  | **Retained** |  |
| 22 | 4 | 0.57 | Relevant - If the patient gives consent.  Very relevant | **Retained** | Pilot survey error – no ability to rate item. Item retained due to written feedback. |
| 23 | 6 | 0.86 |  | **Retained** |  |
| 24 | 7 | 1.00 | This seems a little out of place given location and appointment specifics in previous section. | **Retained** |  |
| 25 | 7 | 1.00 |  | **Retained** |  |
| 26 | 7 | 1.00 |  | **Retained** |  |
| 27 | 7 | 1.00 |  | **Retained** |  |
| 28 | 6 | 0.86 |  | **Retained** | Survey items restructured, to account for written feedback. |
| 29 | 5 | 0.71 | Is this the same as q28. | **Retained** |  |
| 30 | 7 | 1.00 |  | **Retained** |  |
| 31 | 6 | 0.86 |  | **Retained** |  |
| 32 | 7 | 1.00 |  | **Retained** |  |
| 33 | 7 | 1.00 |  | **Retained** |  |
| 34 | 7 | 1.00 |  | **Retained** |  |
| 35 | 6 | 0.86 |  | **Retained** |  |
| 36 | 6 | 0.86 |  | **Retained** |  |
| 37 | 7 | 1.00 |  | **Retained** |  |
| 38 | 7 | 1.00 |  | **Retained** |  |

| **Process - the combinations of the actions that make up the follow-up service delivery** | | | | | |
| --- | --- | --- | --- | --- | --- |
| **Item No.** | **No. of Experts in Agreement** | **Item CVI** | **Free text comments** | **Decision** | **Action(s)** |
| 39 | 7 | 1.00 |  | **Retained** |  |
| 40 | 7 | 1.00 | Isn’t this a repeat of an earlier question? | **Retained** |  |
| 41 | 7 | 1.00 |  | **Retained** |  |
| 42 | 7 | 1.00 |  | **Retained** |  |
| 43 | 7 | 1.00 |  | **Retained** |  |
| 44 | 6 | 0.86 |  | **Retained** |  |
| 45 | 7 | 1.00 | How would a respondent answer this for multiple appointment and how would that be interpreted? | **Retained** |  |
| 46 | 6 | 0.86 |  | **Retained** |  |
| 47 | 7 | 1.00 |  | **Retained** |  |
| 48 | 6 | 0.86 | Feels like a repeat of earlier question. | **Retained** |  |
| 49 | 7 | 1.00 | I feel like this is a repeated question. | **Retained** |  |
| 50 | 7 | 1.00 |  | **Retained** |  |
| 51 | 7 | 1.00 | Does this mean to access outpatient/community services or for a review during the clinic session? | **Retained** |  |
| 52 | 7 | 1.00 |  | **Retained** |  |
| 53 | 5 | 0.71 | Varied options for all sorts of scenarios. | **Retained** | Written feedback identifies the relevancy of item. Discussion with the research team as Item CVI being rated below 0.78. Decision to retain items as they provide data which can be related to the service provision. |
| 54 | 7 | 1.00 |  | **Retained** |  |
| 55 | 7 | 1.00 |  | **Retained** |  |
| 56 | 7 | 1.00 |  | **Retained** |  |
| 57 | 5 | 0.71 |  | **Retained** | Survey items restructured, to account for written feedback |
| 58 | 6 | 0.86 | A repeat of earlier question? | **Retained** |  |
| 59 | 7 | 1.00 |  | **Retained** |  |
| 60 | 6 | 0.86 |  | **Retained** |  |
| 61 | 6 | 0.86 |  | **Retained** |  |

| **Outcomes - the effects of health care** | | | | | |
| --- | --- | --- | --- | --- | --- |
| **Item No.** | **No. of Experts in Agreement** | **Item CVI** | **Free text comments** | **Decision** | **Action(s)** |
| **62** | **6** | **0.86** |  | **Retained** |  |
| **63** | **7** | **1.00** |  | **Retained** |  |
| **64** | **6** | **0.86** |  | **Retained** |  |
| **65** | **6** | **0.86** |  | **Retained** |  |
| **66** | **7** | **1.00** |  | **Retained** |  |
| **67** | **6** | **0.86** |  | **Retained** |  |
| **68** | **5** | **0.71** | Could be combined with previous question. | **Retained** | Survey items restructured, to account for written feedback |
| **69** | **5** | **0.71** | Could be combined with previous question. | **Retained** |  |
