# Supplementary material for: Follow-up after major traumatic injury: a survey of services in Australian and New Zealand public hospitals
Source: BMC Health Serv Res. 2024 May 15;24:630. doi: 10.1186/s12913-024-11105-w (PMC11097478; doi:10.1186/s12913-024-11105-w)
Supplement: Supplementary file 6 — Supplementary Material 6 [file 12913_2024_11105_MOESM6_ESM.pdf]

Survey - Part 1 - Hospital Demographics

Exploration of how follow-up care is provided to major trauma patients throughout Australia and New Zealand

Study Objective
To inform the evaluation of trauma follow-up services, an understanding of the types of trauma follow-up services that are available is first required. Through the data collected in this survey the current landscape of trauma follow-up services will be described and facilitate a greater understanding of what services are available to patients and families. Gaps in trauma follow-up care will be identified including where, how what, and to whom trauma follow-up services are delivered.

Overview
This survey will be sent to hospitals throughout Australia and New Zealand, to explore the context of what and how follow-up care is currently delivered to patients who have suffered major traumatic injury(s). Hospitals have been included if they meet one or more of the following criteria:

- An accredited trauma centre (current or previous) according to the Royal Australasian College of Surgeons;

- A regional trauma centre;

- A trauma service

- Submit data to the Australian or New Zealand Trauma Registry

A participant information form is attached to this survey and can be accessed below.

You are receiving the survey link, as you have been identifed as a 'key person' involved in the delivery of trauma care at your hospital. If you do not wish to participate in the survey, please can you delegate completion of this survey to a team member involved in trauma care at your hospital.

Instructions

This survey contains 50 questions and is divided into four sections outlined below. Please answer each question. Depending upon your answers, questions may be presented in either a sequential order, or you may be directed to proceed to a question later in the survey. Estimated time to complete the survey is 20 - 30 minutes.

(1) Hospital Demographics (Questions 1 to 4 - total 4 questions)

(2) Structure - the context where care is delivered (Questions 1 to 22 - total 22 questions)

(3) Process - the combination of the actions that make up the follow-up service delivery (questions 23 to 42 - total 19 questions)

(4) Outcomes - the effects of health care (questions 43 to 50 - total 7 questions)

[Attachment: "Study1_FINAL_PICF_V1.0_30.10.2022_clean.11.11.2022 (1).pdf"]

Section 1

Hospital Demographics

Q1 - Date Completed: ________________________

Q2 - Name of Hospital: ________________________

Q3 - Patient Population:
- ◯ Adults
- ◯ Paediatrics
- ◯ Both

Q4 - Country:
- ◯ Australia
- ◯ New Zealand

Q4a - State/Territory:
- ◯ New South Wales
- ◯ Victoria
- ◯ Queensland
- ◯ South Australia
- ◯ Western Australia
- ◯ Tasmania
- ◯ Northern Territory
- ◯ Australian Capital Territory

Q4b - Provinces:
- ◯ Auckland
- ◯ New Plymouth
- ◯ Wellington
- ◯ Nelson
- ◯ Cantebury
- ◯ Otago

Survey - Part 2 - Survey Questions

Please complete the survey below.

Thank you!

Section 2

Structure: the context where care is delivered

This section contains 22 questions

Physical characteristics of the trauma centre/hospital

Question	Options
Q1 - What is the type of hospital you work at?	◯ Tertiary Hospital - a hospital that provides tertiary care, which is a level of health care obtained from specialists in a large hospital after referral from the providers of primary and secondary care.<br>◯ Regional Hospital - a hospital that serves a geographic region larger than a single local or rural area.<br>◯ Rural Hospital a small hospital, similar to a district general hospital, but provides health in remote and rural areas.
Q2 - Is your hospital classed as a....	◯ Trauma Centre (currently verified by professional organisation i.e. Royal Australasian College of Surgeons)<br>◯ Trauma Centre (previously verified by professional organisation i.e. Royal Australasian College of Surgeons)<br>◯ Trauma Centre (neither previously or currently verified by professional organisation i.e. Royal Australasian College of Surgeons)<br>◯ None of the above<br>◯ Unsure

Q3 - Which of the following definitions best describes your hospital?	◯ Has facilities that provide the full spectrum of care for the most critically injured, from initial reception and resuscitation to discharge and rehabilitation and provides a higher level of trauma related to research, education, data and quality improvement programs. Provides leadership role to other hospitals within a specific trauma region. (Level 1)<br>◯ Provides comprehensive clincial care for the severly injured to supplement the clinical activities of a Level One trauma hospital. Can be either tertiary or regional. (Level 2)<br>◯ Provides high quality care to non-major level trauma, with the capability of stabilising major trauma patients prior to higher level referral and transfer. It can provide definitive care to a limited number of major trauma patients in concert with the regional Level One hospital. (Level 3)<br>◯ Provides resuscitation and early stabilisation of major trauma patients and their prompt referral and transfer to a higher trauma level hospital. (Level 4)
Q4 - How many in-patient beds does your hospital have?	◯ Up to 49<br>◯ Between 50 to 100<br>◯ Between 101 to 200<br>◯ Between 201 to 300<br>◯ Between 301 to 400<br>◯ Between 401 to 500<br>◯ Over 500<br>◯ Unsure
Q5 - Where is your trauma follow-up service located?	◯ At the same hospital as selected above<br>◯ At a different location separate to the hospital but within the same health service<br>◯ Not a physicial location as service is delivered by soley by telehealth (phone/video)
Q5a - Please estimate the kilometres from the hospital to the trauma follow-up service..	__________________________________
Please proceed to Q9	
Q6 - Does the follow-up service have a designated space?	◯ Yes, located in the outpatients department<br>◯ Yes, located in another location within the hospital<br>◯ No, designated space<br>◯ Other please specify ______
Q7 - Do patients have to pay for parking to attend the follow-up service?	◯ Yes<br>◯ No, parking is free<br>◯ No, they get reimbursed (either fully or partially) by the hospital/follow-up service<br>◯ Unsure
Q8 - Does the follow-up service, health service or other body (insurance or government) provide transport to attend the follow-up appointment?	◯ Yes<br>◯ No<br>◯ Unsure

Q8a - Please select all transport options that are provided so patients can attend the follow-up appointments?	☐ Taxi vouchers<br>☐ Fuel vouchers<br>☐ Public transport vouchers<br>☐ Hospital patient transport<br>☐ Other - please specify ______
System Infrastructure	
Q9 - How is the trauma follow-up service funded?	☐ Part of the trauma operational budget<br>☐ External funding (i.e. insurance)<br>☐ Grant funding/hospital foundation/donations<br>☐ Funding is not provided on a fixed term<br>☐ Not funded<br>☐ Unsure
Q10 - Is the funding for the trauma follow-up service permanent?	◯ Yes<br>◯ No<br>◯ Unsure
Operational Elements	
Q11 - Where does the trauma follow-up service sit in the broader operational structure?	◯ Within outpatients<br>◯ Within the surgical division (Orthopaedics, Neurosurgery, General Surgery, Other)<br>◯ Other please specify ______<br>◯ Unsure<br>(Your halfway through this section)
Q12 - Does the trauma follow-up service have a vision or mission statement?	◯ Yes<br>◯ No<br>◯ Unsure
Q13 - Does the trauma follow-up service have an operational or strategic plan?	◯ Yes<br>◯ No<br>◯ Unsure
Q13a - Is the operational or strategic plan...	◯ Department specific<br>◯ Same as the wider health service<br>◯ Other please specify ______<br>◯ Unsure
Resources - Staff	
Q14 - Is your trauma follow-up service run or implemented by trauma specialists?	◯ Yes<br>◯ No
Q14a - Which specialities runs your trauma follow-up service? Please select all that apply	☐ Orthopaedic specialists<br>☐ Neurosurgical specialists<br>☐ General Surgery specialists<br>☐ Allied Health specialists<br>☐ Other - please specify ______
Q15 - Would you consider the trauma follow-up service to be predominantly:	◯ Nurse-led<br>◯ Doctor led<br>◯ Allied Health led<br>◯ Multi-disciplinary led

Q16 - Who are the staff that regularly work in the trauma follow-up service? Please select all that apply:	☐ Nurse<br>☐ Surgeon<br>☐ Staff specialist<br>☐ Geriatrician<br>☐ Physiotherapist<br>☐ Occupational Therapist<br>☐ Speech Pathology<br>☐ Dietician<br>☐ Rehabilitation Service<br>☐ Social Work<br>☐ Mental Health Services - Psychiatrist, Mental Health Nurses<br>☐ Psychologist<br>☐ Neuropsychologist<br>☐ Pain Management services<br>☐ Orthotics and/or prosthetics<br>☐ Indigenous services<br>☐ Interpreter services<br>☐ Administration/Clerical services<br>☐ Financial support (advice only)<br>☐ Legal support<br>☐ Other please specify ______
Q17 - Do the patients attending the follow-up service regularly see the same health care staff member or team?	◯ Yes, they see the same staff/team members each time<br>◯ No they see different staff/team members<br>◯ It depends upon the needs of the patient i.e., for ongoing pain they see the pain team
Q18 - Do staff receive training (informal or formal) training to work in the follow-up service?	◯ Yes - please specify ______<br>◯ No<br>◯ Unsure
Q19 - Does the follow-up service have permanent staff to deliver the service ensuring a continuous service (i.e., not cancelled in times of staff sickness or annual leave)?	◯ Yes<br>◯ No<br>◯ Unsure
Q20 - Does your hospital have additional follow-up service(s) for trauma patients run by surgical sub-specialities?	◯ Yes<br>◯ No<br>◯ Unsure
Q20a Please select all surgical sub-specialities that apply	☐ Cardiothoracics<br>☐ Neurosurgery<br>☐ Vascular surgery<br>☐ Plastic Surgery<br>☐ ENT<br>☐ Other please specify ______
Q21 - Does your hospital have additional trauma follow-up service(s) run by allied health specialities?	◯ Yes<br>◯ No<br>◯ Unsure
Q21a - Please identify the allied health specialities that provide follow-up service for major trauma patients.	☐ Physiotherapy<br>☐ Occupational Therapy<br>☐ Speech Pathology<br>☐ Dietician<br>☐ Social Work<br>☐ Psychology or Neuropsychology<br>☐ Other please specify ______

Resources - Equipment

Q22 - Please identify all equipment and resources available to the health care staff in delivering the follow-up service:	☐ Electronic Medical Records<br>☐ Medical Records from other hospitals (i.e. a linked statewide system)<br>☐ Access to imaging reports (X-ray, CT, MRI, USS)<br>☐ Access to pathology results<br>☐ Access to reports (imaging, pathology) obtained externally to the hospital/health service<br>☐ Telehealth<br>☐ Physicial examination equipment - stethescope, ottoscope<br>☐ Wound care equipment - dressings, sutures etc<br>☐ Pathology<br>☐ Other please specify ______<br>(Last question in Section 2)
Q22a - If the follow-up service uses telehealth (phone/video) is it...	◯ Phone only<br>◯ Video only<br>◯ Combination of phone and video<br>◯ Other please specify ______
Q22b - If the follow-up services uses video-telehealth, is this via....	☐ Microsoft TEAMS<br>☐ ZOOM<br>☐ Skype<br>☐ Facetime<br>☐ Hospital specific software<br>☐ Other please specify ______<br>(After this question you have completed sections 1 & 2)

Section 3

Process - the combination of the actions that make up the follow-up service delivery

This section contains 19 questions

Service Delivery

Q23 - How is the follow-up service delivered?	◯ In person<br>◯ Telehealth (video/phone)<br>◯ Combination of the above
Q23a - If 'telehealth' is used, is it direct to the patient?	◯ Yes, the patient can access telehealth on their own electronic device<br>◯ No, the patient has to attend a local hospital/GP to access the telehealth<br>◯ None of the above, please specify ______
Q23b - If telehealth is used, where is the health professional located?	◯ In a room occupied only by the trauma follow-up health professional<br>◯ In a shared space with other health professionals<br>◯ No dedicated space

Q24 - Are patients offered a choice of delivery methods to attend the follow-up service?	◯ Yes, all patients offered a choice of attendance methods<br>◯ Yes, but only if patients live outside of the local catchment area<br>◯ Yes, but it is dependent on the reason for the follow-up appointment i.e. wound care is in person only<br>◯ No, there is only one method of attending offered<br>◯ No<br>◯ Unsure
Q25 - Does the follow-up service have protocols or guidelines which outline which delivery method is used and why?	◯ Yes<br>◯ No<br>◯ Unsure<br>(Halfway point)
Q26 - Did the follow-up service delivery method change with COVID-19? Please select all that apply:	☐ Yes, the follow-up service was ceased during lockdown periods<br>☐ Yes, the follow-up service was ceased completely<br>☐ Yes the follow-up service changed to telehealth (phone/video) only<br>☐ Yes, the follow-up service was provided by primary care/GPs<br>☐ Yes, the inclusion criteria to attend the clinic was changed (i.e. multi-trauma only)<br>☐ Other please specify ______<br>☐ No<br>☐ Unsure
Q26a - Have the changes to delivering the follow-up service made during COVID-19 remained?	☐ Yes, the follow-up service has not recommenced<br>☐ Yes the follow-up service routinely incoporates telehealth (phone/video)<br>☐ Yes, the follow-up service is routinely provided by primary care/GPs<br>☐ Yes, the inclusion criteria to attend the clinic was permantly changed<br>☐ Other please specify<br>☐ No<br>☐ Unsure
Q27 - Is the follow-up service part of or in conjunction with a specific follow-up program such as the Trauma Survivor Network?	◯ Yes, the follow-up service includes specific follow-up programs (for eg. Trauma Survivors Network). Please specify ______<br>◯ No, the follow-up service is a stand alone, locally derived service

Service Delivery

Q28 - How are trauma patients selected to attend the follow-up service? Please select all that apply:	☐ All trauma patients are eligible to attend<br>☐ Major trauma patients only (Injury Severity Score > 12)<br>☐ Specific inclusion/exclusion criteria<br>☐ Part of an injury or a treatment pathway (eg. blunt chest trauma)<br>☐ GP referral<br>☐ Indentified by other team members from the wider health organisation<br>☐ Self-referral by the patient<br>☐ Referral by a family member/firned/informal carer<br>☐ Dependent upon the proximity of the follow-up service to the patients residential address<br>☐ All of the above
Q29 - Does the trauma follow-up service incorporate services for families or close others of trauma patients?	○ Yes - please specify the services ______<br>○ No<br>○ Unsure
Q30 - Why are patients asked to attend a follow-up service? Please select all that apply:	☐ Routine or 'check-up' care<br>☐ Specific ongoing care requirement<br>☐ Emotional or psychological support<br>☐ Part of protocolised care<br>☐ Other - please specify ______<br>(Your past the halfway mark of this section)
Q31 - How frequently does the follow-up service run?	○ Daily (Monday to Friday only)<br>○ 7 days per week<br>○ 3-4 times per week<br>○ 1-2 times per week<br>○ Weekly<br>○ Fornightly<br>○ Other please specify ______
Q32 - In general at what timepoints are patients invited for their first appointment?	○ Within 2 weeks of discharge from hospital<br>○ Within 3-4 weeks of discharge from hospital<br>○ Within 5-6 weeks of discharge from hospital<br>○ Within 7-8 weeks of discharge from hospital<br>○ More than 8 weeks after discharge from hospital<br>○ Other - please specify ______
Q33 - Are patients offered more than one trauma follow-up appointment?	○ Yes<br>○ Yes, but it depends on certain criteria ______<br>○ No
Q33a - For patients who require multiple appointments, what is the general reason?	○ Ongoing clinical (physical) requirements<br>○ Ongoing clinical (emotional) requirements<br>○ Combination of both clinical and emotional requirements<br>○ At the patient's request<br>○ At the family/friend/informal carer request<br>○ At the request of primary care (GP etc)
Q34 - Does the follow-up service have a discharge criteria?	○ Yes, please specify ______<br>○ No<br>○ Unsure

Information Flow

Q35 - How are patients notified of the trauma follow-up appointment? Please select all that apply	☐ In person<br>☐ Phone<br>☐ Text message<br>☐ Email<br>☐ Letter<br>☐ Other please specify ______
Q36 - In general, are patients notified of their follow-up appointment....	○ Before they leave hospital<br>○ After they leave hospital
Q36a - If patients are notified of their follow-up appointment after they leave hospital, is this by.... (Please select all that apply)	☐ Phone<br>☐ Text message<br>☐ Email<br>☐ Letter<br>☐ Telehealth<br>☐ Combination of the above<br>☐ Other please specify ______
Q37 - Are family members or close others routinely encouraged to attend follow-up appointments (with the patients consent to do so)?	○ Yes, if they are with the patient when discussion of the appointment occurs<br>○ Yes, they are contacted in addition to the patient<br>○ No, not routinely<br>○ Unsure
Q38 - In general, are patients informed of the reason(s) for the follow-up appointment(s), for example wound care, further investigations (blood tests, imaging)?	○ Yes, if the patient is informed of the appointment in person<br>○ Yes, it is incoprated into the letter, text, email etc<br>○ Yes, but the reason is standard, for example 'you are requires to attend for 'check-up' etc<br>○ No<br>○ Unsure
Health Practitioner Acitvities	
Q39 - What activities occur during a follow-up appointment? Please select all that apply:	☐ Physical examination/assessment<br>☐ Pain assessment<br>☐ Quality of life assessment<br>☐ Mental Health assessment (PTSD/Depression tool)<br>☐ Medication review<br>☐ Imaging review<br>☐ Pathology review<br>☐ Further investigations ordered (imaging/pathology/other)<br>☐ Patient education<br>☐ Family or close others education<br>☐ Emotional support<br>☐ Discussion about the recovery journey (what to expect, timelines etc)<br>☐ Family assessment<br>☐ Repeat appointments scheduled<br>☐ Referrals to/for additional services<br>☐ Other please specify ______
Q39a - Does the follow-up service provide educational or health promotional resources (i.e., printed or online) to patients?	○ Yes<br>○ No<br>○ Unsure

Please select all education and/or health promotion materials that are available:

- ☐ Pain and analgesia
- ☐ Blunt Chest Trauma/Chest injuries
- ☐ Pelvic injuries
- ☐ Splenic/Liver injuries
- ☐ Orthopaedic injuries - Upper limbs
- ☐ Orthopaedic injuries - Lower limbs
- ☐ Traumatic Brain Injury
- ☐ Emotional Health (Mental Health/Social Support)
- ☐ Alcohol and drug
- ☐ Financial support (advice only)
- ☐ Legal Support
- ☐ Other please specify ______

Q39b - Does the follow-up service provide educational or health promotional resources (i.e., printed or online) specifically tailored to families or close others of the patient?

- ◯ Yes
- ◯ No
- ◯ Unsure

Please select all education and/or health promotion materials that are available for family or close others:

- ☐ Pain and analgesia
- ☐ Blunt Chest Trauma/Chest injuries
- ☐ Pelvic injuries
- ☐ Splenic/Liver injuries
- ☐ Orthopaedic injuries - Upper limbs
- ☐ Orthopaedic injuries - Lower limbs
- ☐ Traumatic Brain Injury
- ☐ Emotional Health (Mental Health/Social Support)
- ☐ Alcohol and drug
- ☐ Financial support (advice only)
- ☐ Legal Support
- ☐ Other please specify ______

Q39c - Does the follow-up service have specific referral pathways?

- ◯ Yes
- ◯ No
- ◯ Unsure

Please identify all the referral pathways available:

- ☐ Nurse (general or specialist)
- ☐ Surgeon
- ☐ Geriatrician
- ☐ Physiotherapist
- ☐ Occupational Therapist
- ☐ Speech Pathology
- ☐ Dietician
- ☐ Rehabilitation Service
- ☐ Social Work
- ☐ Mental Health Services - Psychiatrist, Mental Health Nurses
- ☐ Psychologist
- ☐ Neuropsychologist
- ☐ Pain Management services
- ☐ Orthotics and/or prosthetics
- ☐ Indigenous services
- ☐ Interpreter services
- ☐ Administration/Clerical services
- ☐ Financial support (advice only)
- ☐ Legal support
- ☐ Other please specify ______

Q39d - Are the referrals generally.....

- ◯ Located within the hospital or health service where the follow-up service is located
- ◯ Located within another hospital (different health service)
- ◯ Located within the private hospital sector
- ◯ Located within the primary care sector
- ◯ A mixture of the above

Q40 - Does the follow-up service use specific protocols/guidelines/procedures?

- ◯ Yes
- ◯ No
- ◯ Unsure

Q40a - Please identify from the protocols, guidelines and/or procedures that are available in your follow-up service:

- ☐ Pain Management
- ☐ Injury specific (Blunt Chest Trauma, Splenic Injury etc)
- ☐ Psychological and emotional health
- ☐ Readmission to hospital
- ☐ Other please specify ______

Q41 - Does the follow-up service use specific assessment tools for patients or (if applicable) family members/close others?

- ◯ Yes
- ◯ No
- ◯ Unsure

Please go to Section 4, Q43

Q42 - Please select all assessment tools that apply:

		Patient	Family or Close Others	Both	Not applicable/Not used
1	Quality of Life (SF-12/36, EQ-3/5D	◯	◯	◯	◯
	Pain Assessment (Numerical Rating Scale/Brief Pain Inventory)	◯	◯	◯	◯
	PTSD - screening	◯	◯	◯	◯
	PTSD - diagnosis	◯	◯	◯	◯
	Depression - screening	◯	◯	◯	◯
	Depression - diagnosis	◯	◯	◯	◯
	Anxiety	◯	◯	◯	◯
	Mental Health	◯	◯	◯	◯
	Alcohol Use	◯	◯	◯	◯
	Substance Use	◯	◯	◯	◯
	Return to Work	◯	◯	◯	◯
	Cognitive functioning	◯	◯	◯	◯

Section 4

Outcomes - the effects of healthcare

Patient and Family

Q43 - Does the follow-up service evaluate patient satisfaction/experiences with the follow-up care?

- ◯ Yes every patient is asked to complete a satisfaction survey
- ◯ Yes, as part of a wider hopsital or health service survey
- ◯ Yes, occassionally, but not routinely
- ◯ No
- ◯ Unsure

(5 questions to go)

Q44 - Does the service reguarly evaluate family satisfaction/experiences with the follow-up care?

- ◯ Yes, all family members are asked to complete a satisfaction survey
- ◯ Yes, occassionally but not routinely
- ◯ Yes, as part of wider hospital surveys
- ◯ No
- ◯ Unsure

Professional

Q45 - Does the service regularly evaluate staff satisfaction/experiences with the follow-up care?

- ◯ Yes, all staff members are asked to complete a satisfaction survey
- ◯ Yes, occassionally but not routinely
- ◯ Yes, as part of wider hospital surveys
- ◯ No - please go to Q47
- ◯ Unsure - please go to Q47

(Almost finished)

Q46 - How frequently is the results of satisfaction/experiences evaluations reviewed?

	Weekly	Monthly	Every 3 months	Every 6 months	Annually	Unsure	Not applicable
Patients	◯	◯	◯	◯	◯	◯	◯
Family or close others	◯	◯	◯	◯	◯	◯	◯
Staff	◯	◯	◯	◯	◯	◯	◯

Organisational

Q47 - Does the follow-up service have specific Key Performance Indicators (KPIs) in place to measure the service?

- ◯ Yes - please specify ______
- ◯ No
- ◯ Unsure

Q48 - Does the folllow-up service have a procedure to deal with incident reporting?

- ◯ Yes
- ◯ No
- ◯ Unsure

Q49 - Does the follow-up service have a procedure to deal with complaints?

- ◯ Yes
- ◯ No
- ◯ Unsure

End of Survey

Q50 - Please leave any additional comments that you believe mya be helpful in relation to trauma follow-up care

____

Study 2 - Trauma Clinician/Expert Interviews

Please complete the survey below.

Thank you!

Study 2: To explore what and how follow-up care is currently provided to major trauma patients throughout Australia and New Zealand.

In this study an examination and exploration of the experiences of trauma experts and clinicians in delivering trauma follow-up care, will be undertaken. Using semi-structured interviews, understandding what works well and why, which aspects of the service are evaluated and how the quality of follow-up care is measured, will help to provide a deeper understanding of trauma follow-up care.

It is anticipated that the interviews:
- Will occur between June to September 2023
- Will last approximately 1 hour
- Will be conducted either in person, over the phone or via videoconfereencing (TEAMS/ZOOM etc)
- Will occur at a time and date that is conveniant to you, the participant

If you would like any further information please contact:
Elizabeth Wake (PhD Candidate)
liz.wake@griffithuni.edu.au

If you would like to register your interest to participate in this study please click on the Watch Video link below:
